# Supplementation with *Lactobacillus helveticus* NS8 alleviated behavioral, neural, endocrine, and microbiota abnormalities in an endogenous rat model of depression

**DOI:** 10.3389/fimmu.2024.1407620

**Published:** 2024-09-13

**Authors:** Husile Alatan, Shan Liang, Yosuke Shimodaira, Xiaoli Wu, Xu Hu, Tao Wang, Jia Luo, Katsunori Iijima, Feng Jin

**Affiliations:** ^1^ Department of Gastroenterology and Neurology, Akita University Graduate School of Medicine, Akita, Japan; ^2^ Mirai Food Academic Institute of Japan, Akita, Japan; ^3^ Key Laboratory of Mental Health, Institute of Psychology, Chinese Academy of Sciences, Beijing, China; ^4^ Psychology College, Sichuan Normal University, Chengdu, China

**Keywords:** microbiota, mycobiota, serotonin, noradrenaline, corticotropin releasing hormone, gut barrier, inflammation, butyrate

## Abstract

**Introduction:**

Major depressive disorder is a condition involving microbiota–gut–brain axis dysfunction. Increasing research aims to improve depression through gut microbiota regulation, including interventions such as probiotics, prebiotics, and fecal microbiota transplants. However, most research focuses on exogenous depression induced by chronic stress or drugs, with less attention given to endogenous depression. Additionally, research on gut mycobiota in depression is significantly less than that on gut bacteria.

**Methods:**

In the present study, Wistar–Kyoto rats were used as an endogenous depression and treatment-resistant depression model, while Wistar rats served as controls. Differences between the two rat strains in behavior, gut bacteria, gut mycobiota, nervous system, endocrine system, immune system, and gut barrier were evaluated. Additionally, the effects of *Lactobacillus helveticus* NS8 supplementation were investigated.

**Results:**

Wistar–Kyoto rats demonstrated increased depressive-like behaviors in the forced swimming test, reduced sucrose preference in the sucrose preference test, and decreased locomotor activity in the open field test. They also exhibited abnormal gut bacteria and mycobiota, characterized by higher bacterial α-diversity but lower fungal α-diversity, along with increased butyrate, L-tyrosine, and L-phenylalanine biosynthesis from bacteria. Furthermore, these rats showed dysfunction in the microbiota–gut–brain axis, evidenced by a hypo-serotonergic system, hyper-noradrenergic system, defective hypothalamic–pituitary–adrenal axis, compromised gut barrier integrity, heightened serum inflammation, and diminished gut immunity. A 1-month *L. helveticus* NS8 intervention increased the fecal abundance of *L. helveticus*; reduced the abundance of *Bilophila* and Debaryomycetaceae; decreased immobility time but increased climbing time in the forced swimming test; reduced hippocampal corticotropin-releasing hormone levels; decreased hypothalamic norepinephrine levels; increased hippocampal glucocorticoid receptor, brain-derived neurotrophic factor dopamine, and 5-hydroxyindoleacetic acid content; and improved the gut microbiota, serotonergic, and noradrenergic system.

**Conclusion:**

The depressive phenotype of Wistar–Kyoto rats is not only attributed to their genetic context but also closely related to their gut microbiota. Abnormal gut microbiota and a dysfunctional microbiota–gut–brain axis play important roles in endogenous depression, just as they do in exogenous depression. Supplementing with probiotics such as *L. helveticus* NS8 is likely a promising approach to improve endogenous depression and treatment-resistant depression.

## Introduction

Major depressive disorder (MDD) ranks among the most prevalent mental disorders globally (1), yet its specific etiology remains incompletely understood. Gut microbiota dysbiosis and microbiota–gut–brain axis dysfunction may play pivotal roles in the pathophysiology of depression (2–5). MDD patients exhibit different gut microbiota from healthy controls, and transplanting fecal microbiota from patients into germ-free mice or microbiota-depleted rats induces depression in the recipient rodents (6, 7). Conversely, transplanting fecal microbiota from healthy individuals to MDD patients alleviates symptoms (8, 9). Antibiotics treatment can disturb gut microbiota and potentially induce depression (10, 11). Supplementation of specific probiotics can improve MDD symptoms (12, 13). Additionally, prebiotics (14), fermented foods (15), and a healthy diet (16, 17), known to help maintain balanced microbiota, have demonstrated some antidepressant effects. Chronic stress and early life stress, two typical depression risk factors, can also disrupt microbiota and induce microbiota–gut–brain axis dysfunction (18, 19). A poor diet that is high in ultra-processed foods, one of the most common factors disrupting gut microbiota (20), can increase the risk of mental disorders including depression (21).

Targeting gut microbiota to improve mental disorders, including depression, has become a hot point in recent years. Increasing clinical research has shown that probiotic supplementation alleviated symptoms of MDD patients (12, 13). Mechanistic studies have also found that probiotics improve microbiota–gut–brain axis function by promoting brain neurogenesis, regulating neurotransmitter metabolism, improving hypothalamic–pituitary–adrenal (HPA) axis function, exerting anti-inflammatory effects, and reducing gut barrier permeability (22–24). To date, dozens of probiotics have demonstrated antidepressant effects, including species such as *Lactobacillus helveticus*, *Lactobacillus reuteri*, and *Bifidobacterium longum* (22–25). However, most research has concentrated on exogenous depression, with relatively few studies addressing endogenous depression. Despite similar symptoms, their etiologies differ markedly: exogenous depression is triggered by external events or life changes, whereas endogenous depression stems from internal factors such as genetics and biochemical imbalances. Furthermore, most research has focused on the role of gut bacteria, with only two studies investigating gut mycobiota. Both studies identified alterations in gut mycobiota and bacteria–fungi interaction networks in patients with depression (26, 27).

Wistar–Kyoto (WKY) rats, an inbred strain developed from Wistar rats, were initially used as normotensive controls for spontaneous hypertension rats (SHR). They were soon found to exhibit typical depressive symptoms, including psychomotor retardation, learned helplessness, anhedonia, and increased susceptibility to stress-induced ulcers (28, 29). Thus, this unique rat strain is widely utilized as a model for endogenous depression, treatment-resistant depression, and irritable bowel syndrome (IBS) (30, 31).

*L. helveticus* NS8, an isolate from naturally fermented dairy products collected from Inner Mongolia grassland, has demonstrated promising effects in antidepression and regulation of the microbiota–gut–brain axis. NS8 supplementation alleviated depression-related behavioral and gut–brain axis abnormalities in SD rats (24, 32) and regulated the brain peptidome, HPA axis, and gut microbiota in C57BL/6 J mice (33). Additionally, it demonstrated excellent binding capacity to intestinal epithelial cells and anti-inflammatory properties in both *in vivo* and *ex vivo* experiments (24, 32, 34, 35).

To investigate the role of gut microbiota in endogenous depression and whether genetic background affects probiotic intervention, we used WKY rats as the endogenous depression model and Wistar rats as controls. Gut bacteria (16S rRNA sequencing) and gut mycobiota (18S rRNA sequencing) were analyzed to investigate the association between gut microbiota and the depressive phenotype. Depression-like behaviors were evaluated to ascertain differences between the two rat strains and to determine whether *L. helveticus* NS8 exhibited an antidepressant effect. Additionally, indicators of the microbiota–gut–brain axis, including neurotransmitters, cytokines, hormones, neuropeptides, mucins, tight junction proteins, and short-chain fatty acid (SCFA) receptors, were assessed to explore how the microbiota influence behavior.

## Materials and methods

### Animals and treatments

The animal experiments were approved by the Institutional Animal Care and Use Committee of the Institute of Psychology, Chinese Academy of Sciences. Adult male specific-pathogen-free WKY rats and Wistar rats were obtained from Vital River Laboratories (Beijing, China). Rats (weighing 220–240g) were individually housed under standard laboratory conditions throughout the experiment (12/12 h light/dark cycle, lights on at 07:00 h; 22°C–24°C, 40%–60% humidity). The experiments commenced 2 weeks after the rats’ arrival for accommodation.

In experiment 1 (E1), 16 WKY rats were randomly allocated into two groups: a control group and a stress group. Similarly, 16 Wistar rats were also randomly allocated into a control group and a stress group. The stress group were restrained in polypropylene cylinders (6 cm inner diameter, with air vents at the nasal end of the cylinder and length adjusted for each rat) for 6 hours per day for 7 days. Following the stress period, all rats underwent behavioral testing. Fresh fecal samples were collected at the end of the experiment and stored at −80°C until sequencing. The flow diagram was provided in **Supplementary Figure S1A**.

**Figure 1:**
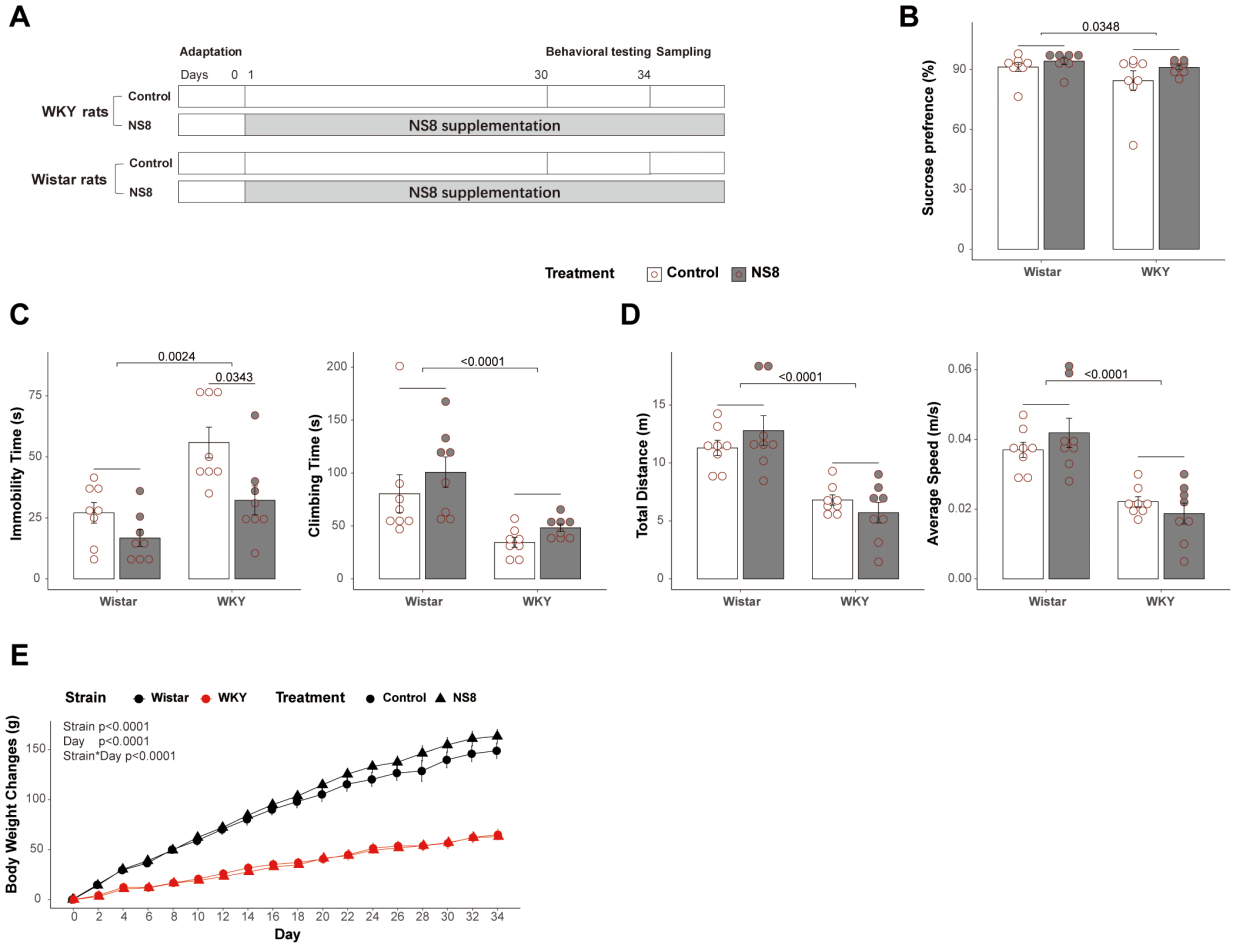
WKY rats presented abnormalities with Wistar rats in behaviors. **(A)** Brief flow diagram of experiment 2. **(B)** Rats’ behaviors in sucrose preference test. **(C)** Immobility time and climbing time in forced swimming test. **(D)** Locomotor activities in open field test. **(E)** Body weight gain of the rats during the experiment 2. Scheirer–Ray–Hare test followed by Dunn’s test adjusted by B–H method was used in parts **(B, C)**, two-way ANOVA followed by Tukey HSD test was used in part **(D)**, and repeated measures ANOVA was used in part **(E)**.

In experiment 2 (E2), 16 WKY rats were randomly allocated into a control group and a *Lactiobacillus* group. Similarly, 16 Wistar rats were also randomly allocated into a control group and a *Lactobacillus* group. The *Lactobacillus* groups received daily supplementation of *L. helveticus* NS8 in their drinking water at a concentration of 10^9^ CFU/ml until the end of the experiment. The drinking water was changed every 24 h to ensure the number of live bacteria not declined lower than 10^8^ CFU/ml. Behavioral testing was performed after 30 days of *L. helveticus* NS8 supplementation. The flow diagram was provided in **Figure 1A**. *L. helveticus* NS8 was cultured, extracted, and suspended using the same procedure as in our previous study (24).

### Behavioral testing

*Open field test (OFT).* An open-field apparatus (50 cm × 50 cm × 50 cm) was used to measure locomotor activity. Rats were placed in the center; their behavior was recorded for 5 min using a CCD camera. The images were recorded on an IBM computer with Image ANYMAZE software (Stoelting, USA). The total distance and mean moving speed were considered indices of locomotor activity.

Forced swimming test (FST). Depressive-like behaviors were evaluated using FST. The forced swim apparatus consisted of polypropylene cylinders (30 cm inner diameter and 50 cm height). During the test, the water depth was approximately 30 cm, and the water temperature was 24°C ± 1°C. The test included a 2-day protocol. On day 1, the rats were placed in the cylinder and allowed to swim for 15 min for adaptation. Twenty-four hours later, the rats were placed in the cylinder again, and their swimming behaviors were recorded for 5 min. The durations of climbing (struggling), swimming, and immobility (floating in the water without movement) were all recorded.

Sucrose preference test (SPT). A modified version of SPT was utilized to evaluate anhedonia in rats according to Chiba (2012) (36). Rats were provided with free access to 200 mL of tap water and 200 mL of a 1% sucrose solution for 24 h. The consumption of tap water and the 1% sucrose solution was recorded. The positions of these bottles were counterbalanced among the rats and switched them once after 12 h. Sucrose preference is expressed as the percentage of 1% sucrose consumption over the total liquid consumption (the sum of 1% sucrose consumption and tap water consumption).

### Animal termination and tissue dissection

After the behavioral tests, the rats were initially rendered unconscious by CO_2_ inhalation, then sacrificed by quickly decapitation, and trunk blood samples were collected into sterile tubes. The tubes were then centrifuged at 3,000 rpm at 4°C for 10 min. The serum was then aspirated and stored at −80°C until further analysis. The whole brains were immediately removed and placed on ice-cold plates. Subsequently, the hippocampus and hypothalamus were rapidly isolated, frozen in dry ice, and stored at −80°C. Samples from the colon and ileum (approximately 3 cm above and below the cecum), and granular feces from the rectum, were also promptly collected, frozen in dry ice, and stored at −80°C for further analysis.

### Enzyme-linked immunosorbent assay analysis

The hippocampus, hypothalamus, ileum, and colon samples were homogenized in phosphate-buffered saline (0.1 mol L^−1^) on ice. The homogenate was then centrifuged at 3,500 rpm for 10 min at 4°C. The supernatants were aspirated and stored at −80°C until further analysis. The contents of monoamine neurotransmitters [including serotonin (5-hydroxytryptamine, 5-HT), 5-hydroxyindoleacetic acid (5-HIAA), norepinephrine (NE), and dopamine (DA)], brain-derived neurotrophic factor (BDNF), glucocorticoid receptor (GR), corticotropin-releasing hormone (CRH), tryptophan hydroxylase 2 (TPH2), indoleamine 2, 3-dioxygenase 1 (IDO1), tyrosine hydroxylase (TH), interleukin-17a (IL-17a), and interleukin-10 (IL-10) in the hippocampal supernatants were determined using ELISA kits following the manufacturer’s instructions. The levels of 5-HT, NE, DA, BDNF, GR, CRH, and corticosterone (CORT) in the hypothalamic supernatants were also determined using ELISA kits. Additionally, levels of 5-HT, 5-HIAA, NE, DA, 4-hydroxy-3-methoxyphenylacetic acid (HVA), TH, gamma-aminobutyric acid (GABA), glutamic acid (GLU), and substance P (SP) in the ileal and colonic supernatants were determined using ELISA kits. Levels of 5-HT, 5-HIAA, adrenocorticotropic hormone (ACTH), CORT, IL-17a, IL-10, interleukin-6 (IL-6), interleukin-1β (IL-1β), tumor necrosis factor-alpha (TNF-α), transforming growth factor-beta (TGF-β), interferon-gamma (IFN-γ), and lipopolysaccharide (LPS) in the serum were also detected using ELISA kits. The GABA and GLU ELISA kits were purchased from Shanghai Enzyme-Linked Biotechnology Co., Ltd. (Shanghai, China), while the others were obtained from Cusabio Biotech Co., Ltd. (Wuhan, China). The sensitivity of each kit is shown in **Supplementary Table S1**.

### Reverse transcriptase-polymerase chain reaction

For RT-PCR analysis, total RNA was extracted from the samples after homogenization and converted to cDNA using standard methods. Colonic and ileal samples were homogenized in 1 ml of TRI Reagent (Thermo Fisher Scientific Inc., Waltham, MA, United States). RNA isolation was performed using traditional methods involving sequential washes with bromochloropropane, isopropanol, and 75% ethanol. RNA concentrations were measured using a NanoDrop Lite Spectrophotometer (Thermo Fisher Scientific Inc., Waltham, MA, United States). Subsequently, the qualified RNA samples were converted to double-stranded cDNA using a TIANScript Reverse Transcription Kit (Tiangen Biotech Co., Ltd., Beijing, China). The collected cDNA samples were subsequently used in quantitative real-time RT-PCR to measure mRNA levels. The sequences of the forward and reverse primers used for amplification were described in **Supplementary Table S2**. The RT-PCR reactions were performed using the ROCHE LightCycler480 (Roche Diagnostics). A standard tissue sample was used to determine the efficiency of the PCR reactions. The housekeeping gene glyceraldehyde-3-phosphate dehydrogenase (GAPDH) was used for internal normalization. Relative mRNA quantification was calculated using the ΔΔCt method.

### Microbiota gene sequencing and bioinformatic analysis

The 16S rRNA and 18S rRNA sequencing of the fecal samples were performed at Wekemo Tech Co., Ltd. (Shenzhen, China). The V3–V4 hypervariable regions of the bacterial 16S rRNA genes were amplified with primers 338F (5′-ACTCCTACGGGAGGCAGCAG-3′) and 806R (5′-GGACTACHVGGGTWTCTAAT-3′) using a thermocycler polymerase chain reaction (PCR) system (GeneAmp 9700, ABI, TX, USA). The fungal 18S rRNA genes were also amplified with primers SSU0817F (5′-TTAGCATGGAATAATRRAATAGGA-3′) and 1196R (5′-TCTGGACCTGGTGAGTTTCC-3′) using the same thermocycler polymerase chain reaction (PCR) system. The qualified samples were sequenced on an Illumina MiSeq platform following the standard protocols at Wekemo Tech Co., Ltd. Amplicon sequence variants (ASVs) were identified using the amplicon denoising algorithm (DADA2). α- and β-diversities were calculated using the core-metrics-phylogenetic plugin in QIIME 2. Microbial functions (MetaCyc pathways) were predicted using the phylogenetic investigation of communities by reconstructing unobserved states (PICRUSt2) based on high-quality sequences. We conducted an in-depth analysis of the sequencing data. The Linear Discriminant Analysis Effect Size (LEfSe) analysis was conducted in Galaxy (usegalaxy.org), the MetaCyc analysis was performed in STAMP 2.1.3 (Dalhousie University), and the correlation network was visualized using Cytoscape 3.10.0 (cytoscape.org).

### Statistical analysis

R version 4.2.3 (R Core Team, 2013) was used to analyze the behavioral, physiological, and microbiota data and to draw figures. Normality and homoscedasticity of the data were assessed using the Shapiro–Wilk test and Levene’s test, respectively. Data showing normal distribution and homogeneity of variances were subjected to two-way analysis of variance (ANOVA) with *post-hoc* Tukey honestly significant difference (Tukey HSD) test. Data that did not meet these criteria were analyzed using the Scheirer–Ray–Hare test, followed by Dunn’s test adjusted by the Benjamini–Hochberg (B–H) method. Correlation analysis was performed using Spearman’s correlation, with subsequent adjustment using the B–H method. Data are presented as mean ± standard error of the mean (SEM). The microbiota data were also analyzed using the aforementioned nonparametric tests. A p-value < 0.05 or adjusted p-value < 0.05 was considered significant.

## Results

### WKY rats presented abnormal behaviors compared with Wistar rats

WKY rats exhibited more depressive-like behaviors compared to Wistar rats in both experiments 1 and 2 (**Figure 1**; **Supplementary Figure S1**; **Supplementary Tables S3**, **S4**). The WKY rats showed significantly less climbing time and more immobility time than Wistar rats in the FST (**Figure 1C**; **Supplementary Figure S1C**), less sucrose preference in the SPT (**Figure 1B**), and shorter moving distance and lower speed than Wistar rats in the OFT (**Figure 1D**; **Supplementary Figure S1D**). The WKY rats also grown slower than Wistar rats (**Figure 1E**). These findings indicate that the WKY rats in the present experiments qualify as an endogenous model of depression, as previously reported (29).

### WKY rats presented an abnormal gut–brain axis compared with Wistar rats

Besides behavioral abnormalities, the WKY showed a series of abnormalities in the gut–brain axis (**Supplementary Table S5**).

**Figure 2:**
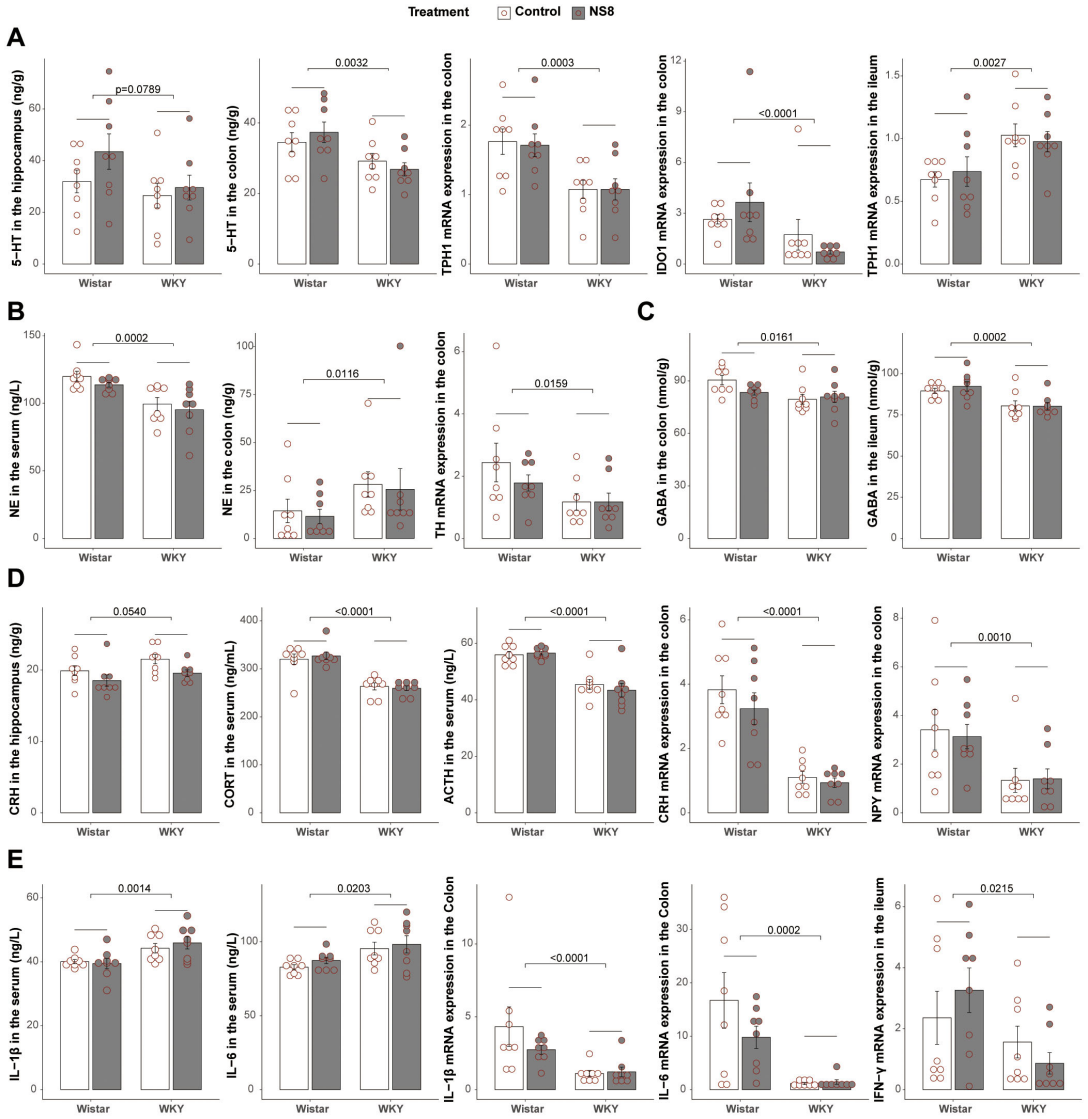
WKY rats presented abnormalities with Wistar rats in brain–gut axis. **(A)** Differences between WKY rats and Wistar rats in serotonergic system. **(B)** Differences in noradrenergic system. **(C)** Differences in GABAergic system. **(D)** Differences in neuroendocrine system. **(E)** Differences in immune system. Two-way ANOVA followed by Tukey HSD test or Scheirer–Ray–Hare test followed by Dunn’s test adjusted by B–H method was used.

First was the serotonergic system (**Figure 2A**). Compared to Wistar rats, WKY rats exhibited lower levels of 5-HT in the hippocampus (marginally significant, p= 0.0789) and colon, reduced mRNA expression of TPH1 and IDO1 in the colon, while increased mRNA expression of TPH1 in the ileum. Next was the noradrenergic system (**Figure 2B**). Compared to Wistar rats, WKY rats exhibited lower levels of NE in the serum but higher concentration in the colon. However, the expression of TH, the rate-limiting enzyme of NE synthesis, was decreased in the colon. The third was the GABAergic system (**Figure 2C**). WKY rats had lower GABA levels than Wistar rats in both the colon and ileum. The fourth was the HPA axis and neuropeptides (**Figure 2D**). Compared to Wistar rats, WKY rats had higher levels of CRH in the hippocampus, lower levels of ACTH and CORT in the serum, and decreased expression of CRH and NPY in the colon. The fifth was the immune system (**Figure 2E**; **Supplementary Table S5**). In serum, WKY rats exhibited lower levels of TGF-β but higher levels of IL-1β, IL-6, and TNF-α than Wistar rats, indicating a higher circulating inflammatory level. In the colon, WKY rats exhibited lower mRNA expression of IFN-γ, IL-1β, IL-6, TNF-α, IL-10, and IL-17a. In the ileum, WKY rats showed lower mRNA expression of IFN-γ and IL-10, both indicating a lower immune activation in the gut of WKY rats.

### WKY rats exhibited a compromised gut barrier compared to Wistar rats

**Figure 3:**
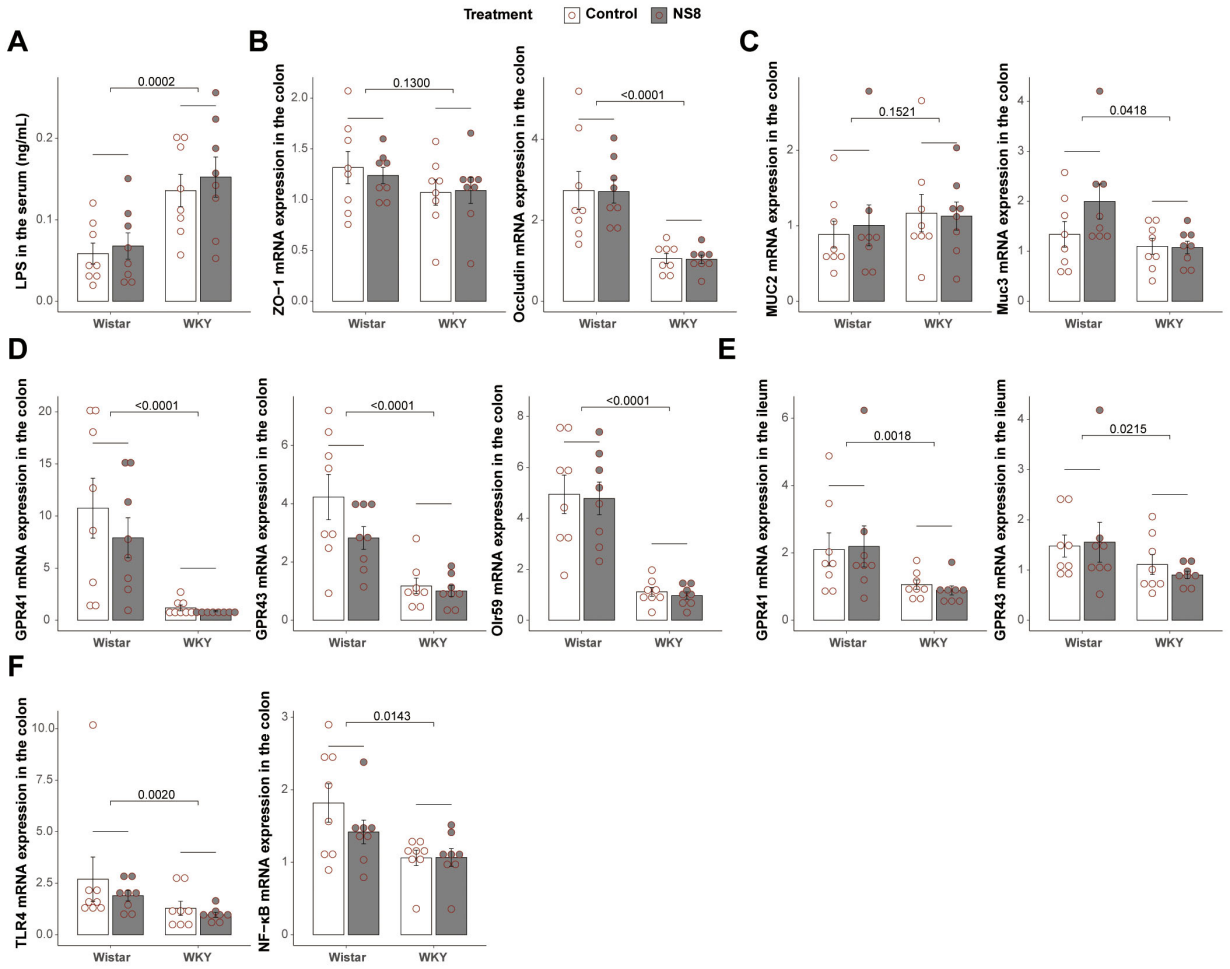
WKY rats presented compromised gut barrier with Wistar. **(A)** Concentration of LPS in the serum. **(B)** mRNA expression of ZO-1 and occludin in the colon. **(C)** Mucin expression in the colon. **(D)** mRNA expression of GPR41, GPR43, and Olr59 in the colon. **(E)** mRNA expression of GPR41 and GPR43 in the ileum. **(F)** mRNA expression of TLR4 and NF-κB in the colon. Two-way ANOVA followed by Tukey HSD test or Scheirer–Ray–Hare test followed by Dunn’s test adjusted by B–H method was used.

Compared to Wistar rats, WKY rats had more than twice the amount of LPS in the serum (**Figure 3A**), indicating higher gut barrier permeability in WKY rats. Regarding tight junctions (TJs, **Figure 3B**), WKY rats had reduced mRNA expression of occludin in the colon, although the reduction in zonula occludens 1 (ZO-1) was not significant. For mucins (**Figure 3C**), WKY rats presented lower mRNA expression of Muc3 in the colon than Wistar rats. These findings indicate compromised gut barrier integrity in WKY rats.

Considering the important role of SCFAs in the microbiota–gut–brain axis (37) and mucosal integrity (38), the mRNA expression of SCFA receptors in the gut was also analyzed. WKY rats exhibited lower mRNA expression of Olr59 (the functional ortholog of Olfr78 in murine and OR51E2 in humans), GPR41, and GPR43 in the colon and lower mRNA expression of GPR41 and GPR43 in the ileum (**Figures 3D, E**). Additionally, the mRNA expression of NF-κB and TLR-4 was decreased in the colon of WKY rats (**Figure 3F**).

### WKY rats exhibited abnormal gut bacteria and mycobiota compared to Wistar rats

**Figure 4:**
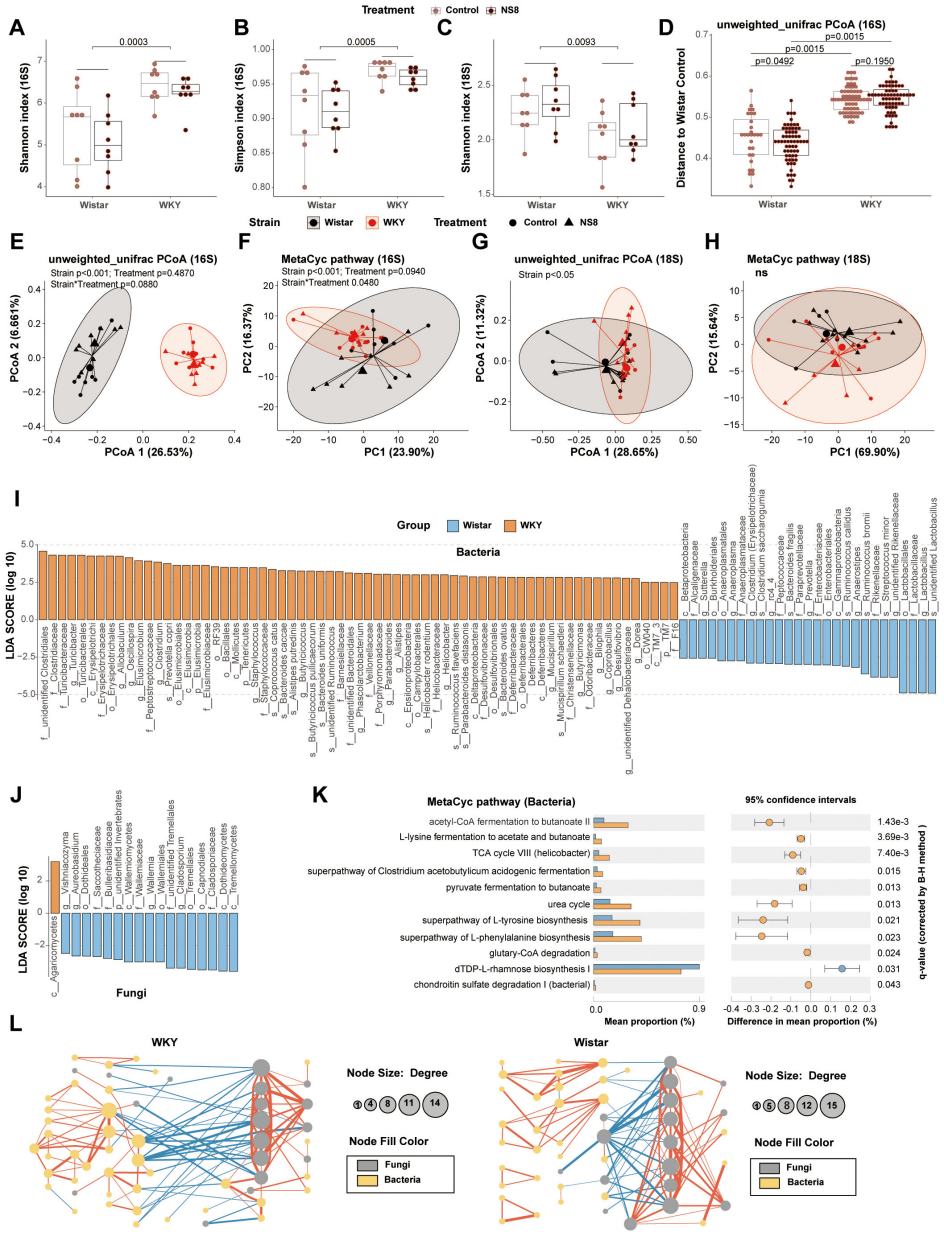
WKY rats presented abnormalities with Wistar rats in gut bacteria and mycobiota. **(A, B)** α-Diversity indexes of bacteria and **(C)** α-diversity indexes of fungi in experiment two. **(D, E)** Principal coordinates analysis (PCOA) of bacteria and **(F)** principal component analysis (PCA) of the predicted function of bacteria. **(G)** PCOA of fungi and **(H)** the PCA of the predicted function of fungi. **(I)** Biomarker bacteria identified by LEfSe analysis between WKY rats and Wistar rats (LDA>2, p<0.05). **(J)** Biomarker fungi between WKY rats and Wistar rats (LDA>2, p<0.05). **(K)** Biomarker MetaCyc pathways identified by STAMP between WKY rats and Wistar rats (Welch’s t-test adjusted by B–H method). **(L)** Bacteria–fungi co-abundance network in WKY rats and Wistar rats. Spearman’s correlations were used and only the adjusted p-value (**B–H** method) < 0.05 are shown. The blue lines are negative correlations, while the red lines are positive correlations. The thicker the line, the greater the absolute value of the correlation coefficient. In parts **(I–L)**, Wistar rats (16 rats, composed of the Wistar control rats in experiments 1 and 2) were compared with the WKY rats (16 rats, composed of the Wistar controls in the two experiments).

WKY rats exhibited distinct gut bacteria and mycobiota compared to Wistar rats in both experiments 1 and 2 (**Supplementary Tables S3**, **S4**). Regarding bacteria, WKY rats had higher α-diversity, indicated by higher Chao1 estimator, Faith’s phylogenetic diversity, Shannon index, and Simpson index, than Wistar rats (**Figures 4A, B**). However, in terms of mycobiota, WKY rats exhibited lower α-diversity, indicated by a lower Shannon index, than Wistar rats (**Figure 4C**). Unweighted-unifrac principal coordinates analysis (PCOA) of bacteria clearly separated the two rat strains (**Figures 4D, E**; **Supplementary Figures S2A, E**), with significant differences observed in predicted bacterial functions [**Figure 4F**; **Supplementary Figure S2B**, principal component analysis (PCA) of MetaCyc]. Fungal PCOA also distinguished the two rat strains (**Figure 4G**; **Supplementary Figure S2C**), but the predicted fungal functional differences were not consistent across the two experiments (**Figure 4H**; **Supplementary Figure S2D**, PCA of MetaCyc).

The LEfSe analysis of bacteria was conducted at the species level and identified 94 biomarker bacteria distinguishing WKY and Wistar rats (**Figure 4I**). WKY rats had a higher abundance of bacteria in 17 families (including Clostridiaceae, Turicibacteraceae, Erysipelotrichaceae, Peptostreptococcaceae, Elusimicrobiaceae, Staphylococcaceae, Barnesiellaceae, Veillonellaceae, Porphyromonadaceae, Helicobacteraceae, Desulfovibrionaceae, Deferribacteraceae, Christensenellaceae, Odoribacteraceae, F16, unidentified Clostridiales family, and unidentified Bacteroidales family) and in the 18 genera (including *Turicibacter*, *Allobaculum*, *Oscillospira*, *Elusimicrobium*, *Clostridium*, *Staphylococcus*, *Butyricicoccus*, *Phascolarctobacterium*, *Parabacteroides*, *Alistipes*, *Helicobacter*, *Mucispirillum*, *Butyricimonas*, *Bilophila*, *Coprobacillus*, *Desulfovibrio*, unidentified *Dehalobacteriaceae* genus, and *Dorea*). Wistar rats had a higher abundance of bacteria in seven families (including Lactobacillaceae, Rikenellaceae, Enterobacteriaceae, Paraprevotellaceae, Peptococcaceae, Anaeroplasmataceae, and Alcaligenaceae) and in the eight genera [including *Lactobacillus*, unidentified *Rikenellaceae* genus, *Anaerostipes*, *Prevotella* (Paraprevotellaceae), rc4_4, *Clostridium* (Erysipelotrichaceae), *Anaeroplasma*, and *Sutterella*]. The LEfSe analysis of mycobiota was conducted at the genus level and identified 18 biomarker fungi (**Figure 4J**).

The STAMP analysis identified 11 biomarker MetaCyc pathways distinguishing WKY and Wistar rats (**Figure 4K**). Four of them belonged to butyrate biosynthesis and were abundant in WKY rats, including acetyl-CoA fermentation to butanoate II (PWY-5676), L-lysine fermentation to acetate and butanoate (P163-PWY), superpathway of *Clostridium acetobutylicum* acidogenic fermentation (PWY-6590), and pyruvate fermentation to butanoate (CENTFERM-PWY). Six other pathways, including the superpathway of L-tyrosine biosynthesis (PWY-6630), superpathway of L-phenylalanine biosynthesis (PWY-6628), TCA cycle VIII (*Helicobacter*) (REDCITCYC), urea cycle (PWY-4984), glutaryl-CoA degradation (PWY-5177), and dTDP-L-rhamnose biosynthesis I (DTDPRHAMSYN-PWY), were also abundant in WKY rats. The only pathway abundant in Wistar rats was chondroitin sulfate degradation I (bacterial) (PWY-6572).

The gut–brain axis biomarker-related pathways were also analyzed (**Supplementary Figures S2F–O**). The biosynthesis of L-tryptophan (precursor of 5-HT synthesis) from bacteria (the sum of TRPSYN-PWY and PWY-6629) showed no difference between the two rat strains. The biosynthesis of L-glutamate and L-glutamine (PWY-5505) from bacteria also showed no difference. However, the biosynthesis of L-tyrosine and L-phenylalanine (PWY-6630 and PWY-6628), the two precursors of NE synthesis, from gut bacteria, were both increased in WKY rats. The butyrate biosynthesis of bacteria (fermentation to butanoate, including P162-PWY, P163-PWY, PWY-5022, PWY-5676, PWY-5677, and CENTFERM-PWY) and the propionate biosynthesis of bacteria (fermentation to propanoate, including P108-PWY, PWY-5088, and PWY-7013) were both increased in WKY rats. However, the biosynthesis of acetate (fermentation to acetate, including P124-PWY, P161-PWY, P162-PWY, P163-PWY, P461-PWY, and PWY-5100) and lactate (fermentation to lactate, including P122-PWY, P124-PWY, P461-PWY, P461-PWY, and ANAEROFRUCAT-PWY) were not affected by treatment or strain. There were no significant differences between the two strains in LPS biosynthesis (the sum of PWY-6467, PWY0-1338, KDO-NAGLIPASYN-PWY, NAGLIPASYN-PWY, and LPSSYN-PWY) and peptidoglycan biosynthesis (the sum of PWY-5265, PWY-6470, PWY-6471, PWY-6385, PWY0-1586, and PEPTIDOGLYCANSYN-PWY).

The bacteria–fungi co-abundance network differed between the two strains (**Figure 4L**). Compared to Wistar rats, WKY rats had more correlations, both negative and positive, between bacteria and mycobiota.

### Thirty days of *L. helveticus* NS8 intervention alleviated the abnormalities in WKY rats’ behaviors and gut–brain axis

**Figure 5:**
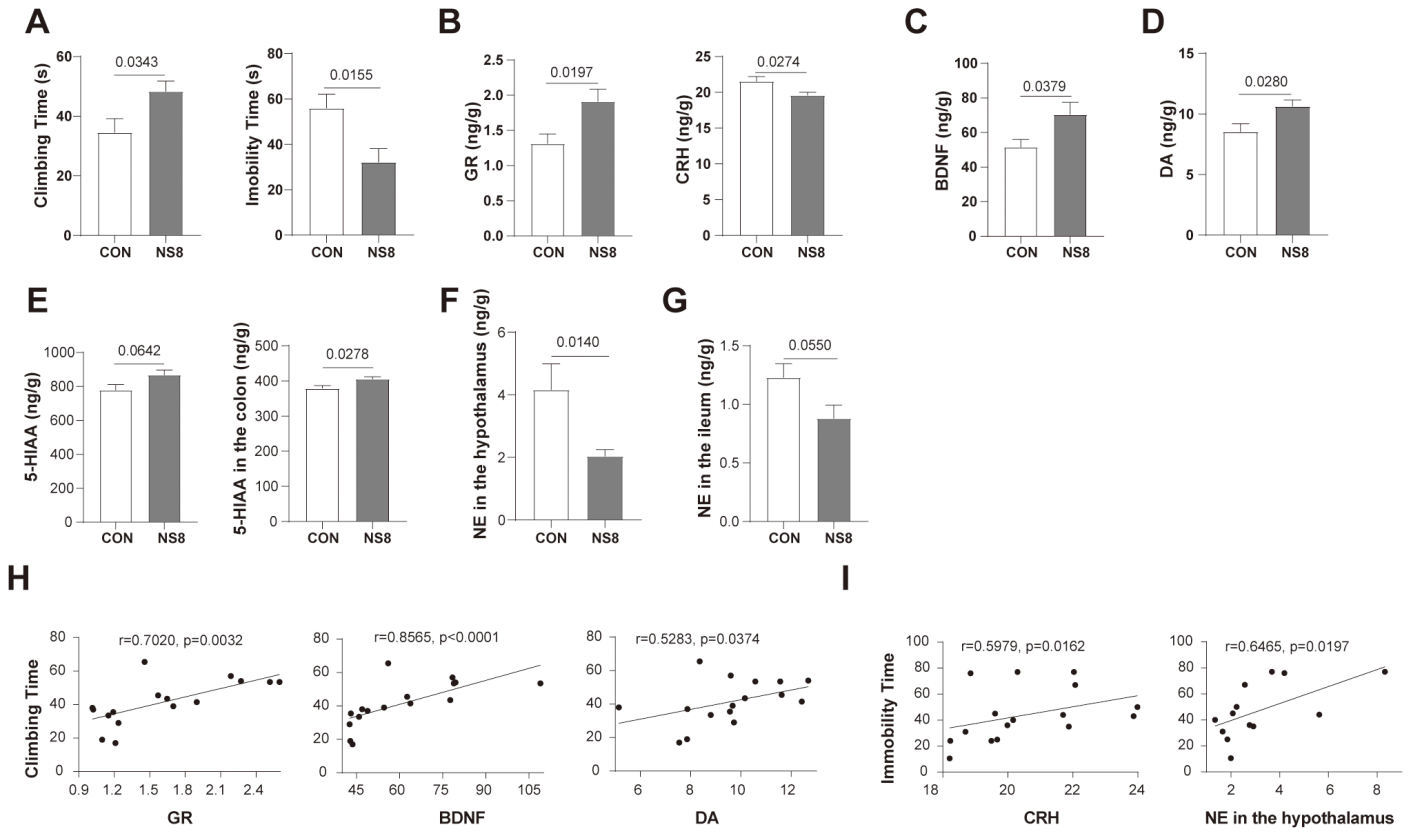
NS8 intervention improved the behavioral and physiological abnormabilities of WKY rats. **(A)** Behaviors of rats in forced swimming test. **(B)** GR and CRH content in hippocampus. **(C, D)** BDNF and DA content in the hippocampus, respectively. **(E)** The 5-HIAA concentration in the hippocampus and colon. **(F, G)** NE concentration in the hypothalamus and ileum, individually. **(H)** Physiological indicators correlated with climbing time. **(I)** Physiological indicators correlated with immobility time. Except for 6–8 rats/group in part **(F)**, the others were all 8 rats/group. Data showing normal distribution and homogeneity of variances were subjected to independent samples t-test **(A, B, D, E, G)**, and the others were subjected to Mann–Whitney U-test (C, F). Spearman’s correlation was used in parts **(H, I)**.

One week of restraint stress inhibited locomotor activity in the OFT (marginally significant, p<0.08, **Supplementary Figure S1D**; **Supplementary Table S3**), while 30 days of NS8 intervention did not change locomotor activity (**Figure 1D**; **Supplementary Table S4**). However, NS8 intervention markedly reduced depressive-like behaviors in the FST. It significantly reduced immobility time and increased climbing time in WKY rats (**Figure 5A**), although the improvement in sucrose preference did not reach a significant level (**Figure 1B**; **Supplementary Table S4**).

Apart from behaviors, NS8 intervention increased GR content in the hippocampus while decreasing CRH content (**Figure 5B**) and increased BDNF and DA concentrations in the hippocampus (**Figures 5C, D**). Furthermore, NS8 intervention raised the content of 5-HIAA in the hippocampus and colon (**Figure 5E**) and decreased the concentration of NE in the hypothalamus and ileum (**Figures 5F, G**). The behavioral improvements due to NS8 intervention were associated with the aforementioned gut–brain axis indicators. The climbing time of WKY rats in the FST was positively correlated with the content of GR, BDNF, and DA in the hippocampus (**Figure 5H**). Additionally, the immobility time was positively correlated with the CRH content in the hippocampus and NE content in the hypothalamus (**Figure 5I**).

### NS8 intervention had a different impact on gut microbiota compared to restraint stress

**Figure 6:**
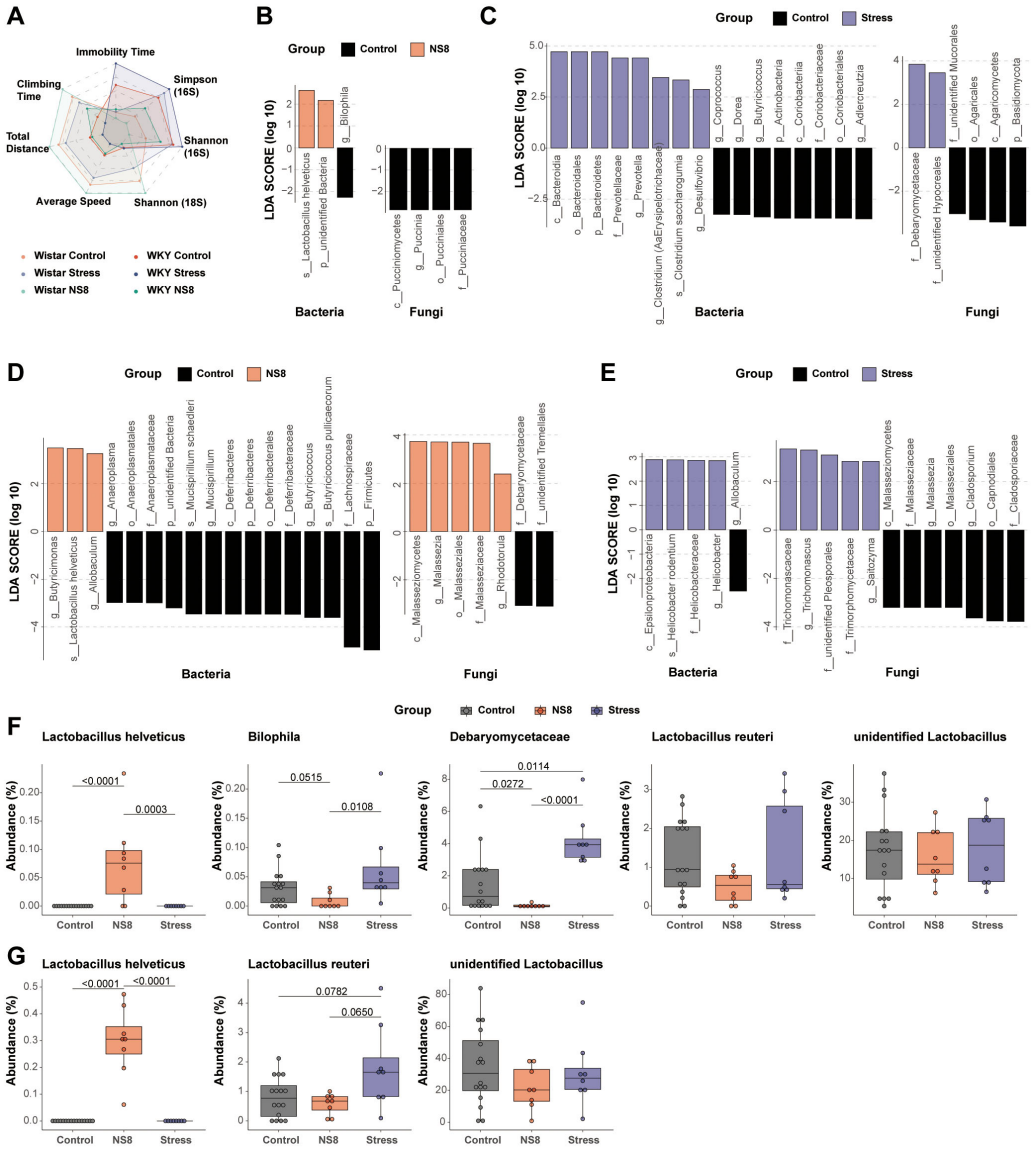
NS8 made opposite effects with restraint stress. **(A)** The influence of the above two interventions on behaviors and α-diversity; different groups were compared with Wistar control as the reference group. **(B)** Effects of NS8 intervention on gut microbiota and **(C)** effects of restraint stress on gut microbiota in WKY rats (LEfSe analysis, LDA>2, p<0.05). **(D)** Effects of NS8 intervention on gut microbiota in Wistar rats and **(E)** effects of restraint stress on gut bacteria and mycobiota in Wistar rats (LEfSe analysis, LDA>2, p<0.05). **(F)** Impaction of the two interventions on *L. helveticus*, *Bilophila*, Debaryomycetaceae, *L. reuteri*, and unidentified *Lactobacillus* in WKY rats. **(G)** Impaction of the two interventions on *L. helveticus*, *L. reuteri*, and unidentified *Lactobacillus* in Wistar rats. Kruskal–Wallis’s test followed by Dunn’s test adjusted by B–H method was used in parts **(F, G)**.

Neither NS8 intervention nor restraint stress affected the α-diversity of bacteria and mycobiota. Restraint stress did not affect the β-diversity, whereas NS8 intervention did. NS8 intervention significantly altered the bacterial β-diversity in Wistar rats but had a less pronounced effect in WKY rats (**Figure 4D**). By using the behaviors and α-diversity indexes as indicators, distinct groups were successfully separated on the radar map (**Figure 6A**). The WKY rats exhibited distinct behaviors and microbiota phenotypes compared with Wistar rats. Seven days of restraint stress imposed a mild depression-inducing effect, whereas NS8 intervention showed a distinct antidepressive effect.

NS8 intervention and restraint stress influenced bacterial and mycobiota compositions differently in both WKY rats (**Figures 6B, C**) and Wistar rats (**Figures 6D, E**). *L. helveticus* was the only biomarker bacterium that increased in both Wistar and WKY rats after NS8 intervention. NS8 intervention reduced the abundance of *Bilophila* and Debaryomycetaceae, while restraint stress raised Debaryomycetaceae’ abundance in WKY rats.

*Lactobacillus* is among the most crucial beneficial bacteria in the mammalian gut. Wistar rats and WKY rats both had two main types of lactobacilli species: unidentified *Lactobacillus* genus and *L. reuteri* (**Figures 6F, G**). The former was present in all rats of both experiments, whereas the latter was present in over 75% of the rats. However, *L. helveticus* did not appear in either strain of rats initially. After 1 month of NS8 supplementation, *L. helveticus* was found in 100% of Wistar rats’ feces but only 75% of WKY rats’ feces (six out of eight rats). NS8 intervention did not make significant effect both in the abundance of unidentified *Lactobacillus* genus and *L. reuteri*, while restraint stress increased the abundance of *L. reuteri* in Wistar rats (marginally significant, p=0.0782).

### The depressive behavioral and physiological phenotypes were correlated with gut microbiota

**Figure 7:**
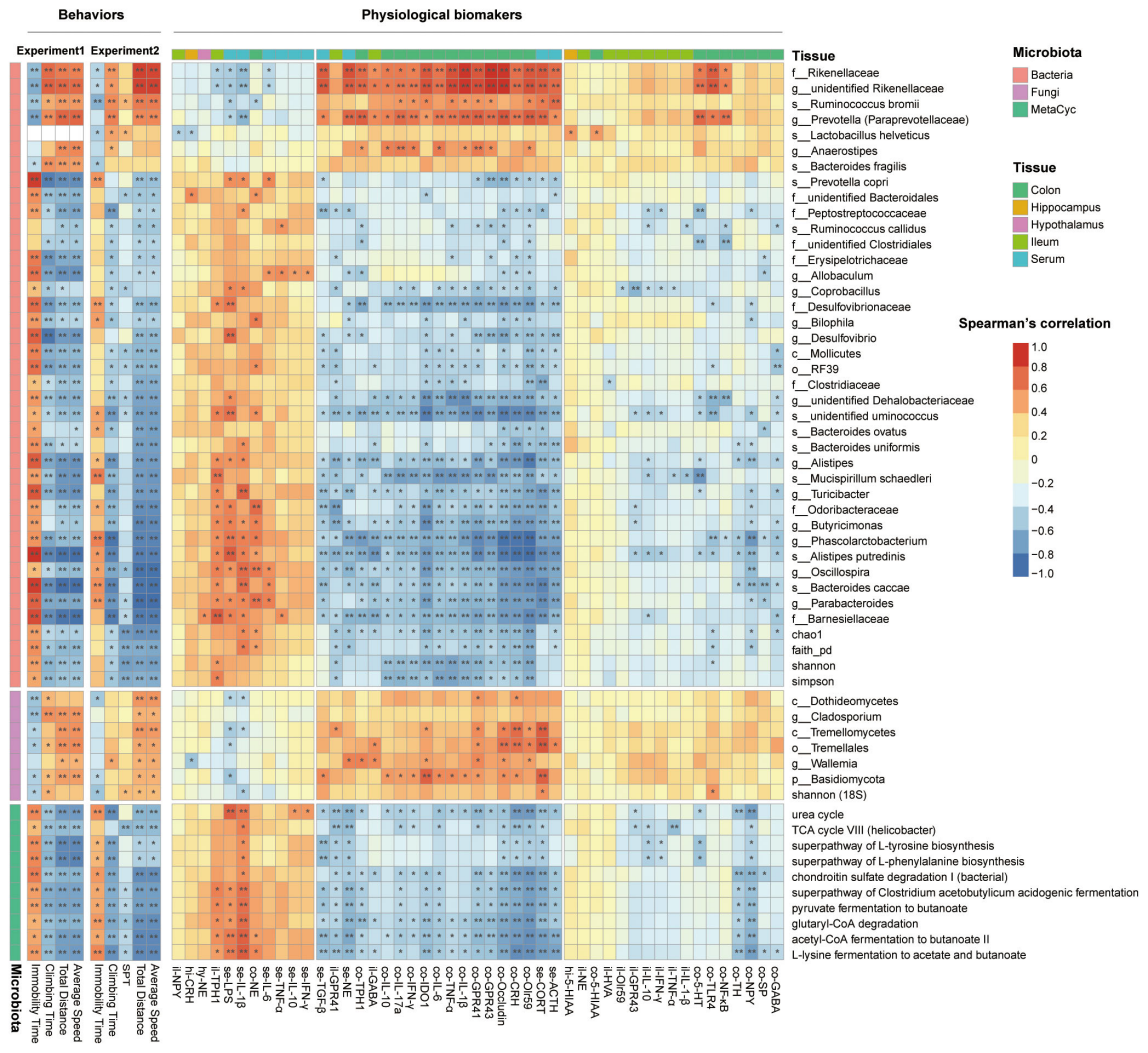
The gut microbiota was correlated with behaviors and physiological indicators. The heatmap showed the correlation between behaviors with microbiota biomarkers and physiological biomarkers with microbiota biomarkers. Spearman’s correlations were used, and only the adjusted p-value (B–H method) are shown. *p.adj < 0.05, **p.adj < 0.01.

For all the biomarker microbe indicators identified, only the lowest taxonomic level was retained if a microbe had duplicate values at different taxonomic levels. The correlation analysis was conducted between the remaining 82 microbes and behaviors in experiments 1 and 2, and 42 microbes that were significantly correlated with behaviors in both experiments were selected. A total of 10 biomarker MetaCyc pathways and five biomarker α-diversity indexes that were significantly correlated with behaviors in both experiments were also used for further analysis (**Figure 7**). In both experiments, the fecal bacteria α-diversity index (including Shannon index, Simpson index, Chao1 index, and Faith’s phylogenetic diversity) was positively correlated with depression-like behaviors. Higher bacterial α-diversity indexes were associated with more immobility time, less climbing time in FST, less sucrose preference in SPT, shorter moving distance in OFT, and lower speed in OFT. On the contrary, the fungal α-diversity index (Shannon index) was negatively correlated with depression-like behaviors. Higher fungal α-diversity indexes were associated with less immobility time, more climbing time in FST, more sucrose preference in SPT, further moving distance in OFT, and higher speed in OFT.

Eight bacteria were negatively correlated with depression-like behaviors, including Rikenellaceae, unidentified Rikenellaceae genus, *Ruminococcus bromii*, Prevotella (Paraprevotellaceae), *L. helveticus*, *Anaerostipes*, *Bacteroides fragilis*, and *Prevotella copri*. A total of 28 bacteria were positively correlated with depression-like behaviors, including Barnesiellaceae, Parabacteroides, Bacteroides caccae, Oscillospira, *Alistipes putredinis*, Phascolarctobacterium, Butyricimonas, Odoribacteraceae, Turicibacter, *Mucispirillum schaedleri*, Alistipes, *Bacteroides uniformis*, *Bacteroides ovatus*, unidentified *Ruminococcus* genus, unidentified *Dehalobacteriaceae* genus, Clostridiaceae, RF39, Mollicutes, Desulfovibrio, *Bilophila*, Desulfovibrionaceae, Coprobacillus, Allobaculum, Erysipelotrichaceae, unidentified Clostridiales family, *Ruminococcus callidus*, Peptostreptococcaceae, and unidentified Bacteroidales family. Six fungi were negatively correlated with depression-like behaviors, including Dothideomycetes, Cladosporium, Tremellomycetes, Tremellales, Wallemia, and Basidiomycota. A total of 10 MetaCyc pathways were all positively correlated with depression-like behaviors.

The physiological indicators were additionally associated with the gut microbiota composition and bacterial function. The abundance of *L. helveticus* was negatively correlated with the CRH content in the hippocampus and NPY mRNA expression in the ileum and positively correlated with the 5-HIAA content in the hippocampus and colon. The abundance of *Bilophila* was negatively correlated with colonic IDO1, CRH, NPY, IL-1, Olr59, and occludin expression, and serum NE content, and positively correlated with colonic NE content. The abundance of Rikenellaceae, unidentified *Rikenellaceae* genus, and *Prevotella* (Paraprevotellaceae) were positively correlated with the content of colonic proinflammatory cytokines, gut barrier permeability indicators, SCFAs receptors, serum stress hormones, TLR-4, NF-κB, and 5-HT, and were negatively correlated with serum inflammation. The abundance of Tremellomycetes and Tremellales were both positively correlated with the colonic gut barrier permeability indicators and SCFAs receptors.

In contrast, the abundance of Barnesiellaceae, *Parabacteroides*, *Bacteroides caccae*, *Oscillospira*, *Alistipes putredinis*, *Phascolarctobacterium*, *Butyricimonas*, Odoribacteraceae, *Turicibacter*, *M. schaedleri*, *Alistipes*, unidentified *Ruminococcus* species, unidentified *Dehalobacteriaceae* genus, and *Desulfovibrionaceae* were negatively correlated with colonic proinflammatory cytokines, gut barrier permeability indicators, SCFAs receptors, serum stress hormones, TLR-4, NF-κB, and 5-HT, and positively correlated with serum inflammation. Acetyl-CoA fermentation to butanoate II, L-lysine fermentation to acetate and butanoate, superpathway of *Clostridium acetobutylicum* acidogenic fermentation, pyruvate fermentation to butanoate, and TCA cycle VIII (*Helicobacter*) presented similar correlations with the above bacteria. Superpathways of L-tyrosine biosynthesis and L-phenylalanine biosynthesis were also negatively correlated with colonic gut barrier permeability indicators and stress hormones.

The physiological indicators were significantly interconnected (**Supplementary Figure S3**). The LPS levels in the serum showed multiple significant negative correlations. Specifically, it was negatively correlated with the levels of NE, ACTH, and CORT in the serum; the mRNA expression of GPR41 in the ileum; and the mRNA expression of NPY, CRH, occludin, Olr59, CFR41, and GPR43 in the colon. The mRNA expression of TPH1 in the ileum showed a negative correlation with the TGF-β content in the serum, the mRNA expression of IDO1, occludin, and CFR41 in the colon, and the mRNA expression of IL-10 in the ileum. The gut barrier biomarkers (occludin, Olr59, GPR41, and GPR43) demonstrated a positive correlation with the inflammatory biomarkers in the colon (including IL-1β, IL-6, IL-10, TNF-α, IL-17a, and IFN-γ). The indicators of neural (5-HT content and mRNA expression of TPH1 and IDO1 in the colon), neuroendocrine (mRNA expression of CRH and NPY in the colon, content of ACTH and CORT in the serum), immune (mRNA expression of IL-1β, IL-6, and IL-17a in the colon), and gut barrier indicators (Occludin, Olr59, GPR41, and GPR43) were positively correlated with each other.

## Discussion

**Figure 8:**
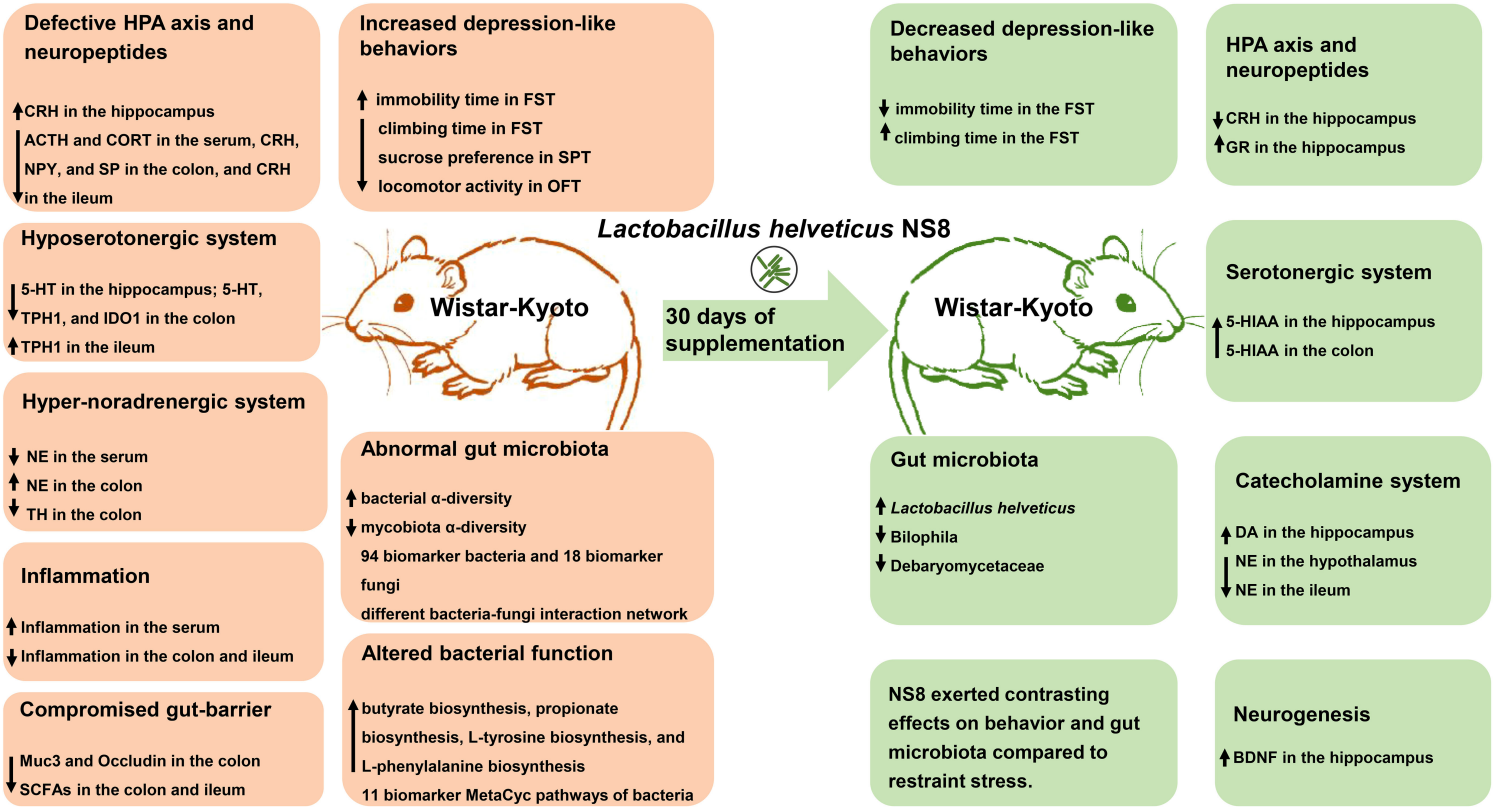
Graphic abstract. The reddish section on the left illustrates the behavioral, microbiota, and microbiota–gut–brain axis abnormalities in WKY rats, while the green section represents the improvements observed after 1 month of NS8 intervention.

In the present study, WKY rats exhibited more depression-like behaviors, including less climbing time and more immobility time in the FST, reduced sucrose preference in the SPT, and decreased locomotor activities in the OFT, as previously reported (28, 29, 39). Moreover, we were the first to find that these endogenous depressive rats had an abnormal microbiota–gut–brain axis. The WKY rats demonstrated higher bacterial α-diversity and lower fungal α-diversity, a different bacteria–fungi interaction network, distinct bacterial function, a compromised gut barrier, fewer SCFA receptors in the ileum and colon, heightened serum inflammation, diminished gut immunity, and abnormalities in the serotonergic system, noradrenergic systems, GABAergic system, and HPA axis. One month of NS8 supplementation alleviated the depression-like behaviors of WKY rats; improved gut microbiota composition and function; reduced the levels of CRH in the hippocampus; increased the levels of GR, DA, and BDNF in the hippocampus; and alleviated the abnormalities in the serotonergic system and noradrenergic systems (**Figure 8**, graphic abstract). The results were in accordance with our previous study. A 3-week intervention with *L. helveticus* NS8 corrected the behavioral abnormalities of exogenous depressive SD rats and improved gut–brain axis function through neural, endocrine, and immune pathways (24).

### Endogenous depression, gut microbiota, and *L. helveticus* NS8

Besides the increased bacterial α-diversity and decreased fungal α-diversity in WKY rats, we also found a positive correlation between depressive-like behaviors and bacterial α-diversity, and a negative correlation between depressive-like behaviors and fungal α-diversity. Although an increasing amount of research confirms the link between depression and the gut microbiota, previous studies have shown inconsistent results regarding α-diversity (40–42). Some studies found that MDD patients had higher bacterial α-diversity than healthy controls, while other studies found lower bacterial α-diversity; however, many studies did not find any differences (42). Additionally, research focusing on gut mycobiota is much less than on bacteria; Ruan et al. (2020) found that patients with a current depressive episode had lower fungal α-diversity than healthy controls (27), while Hao et al. (2023) found no difference between depressive children and adolescents and controls (26). The heterogeneous genetic context could be the reason for these discrepancies, since the host genetic context partially regulates the gut microbiota (43). NS8 intervention had no significant effect on α-diversity, consistent with other research on probiotic intervention in depressive patients (44) and in WKY rats (45).

On the other hand, most research have shown altered bacterial β-diversity in individuals with MDD (42), which was also observed in WKY rats in this study. However, existing research on the specific gut microbiota composition of depressive individuals has not reached a consensus (40–42, 46–49). The biomarker bacteria identified in this study appeared in previously reported lists, but the trends were not identical. Bacteria that were higher in WKY rats, such as *Turicibacter* and *Alistipes*, also increased in MDD patients (41, 50). *Turicibacter* was the only genus consistently decreased in stressed depressed animals (46) but increased in WKY rats (51). Overall, most biomarker bacteria identified in this study did not reach a consensus in previous research. Furthermore, the abundance of certain bacteria at the family level contradicted findings at the genus/species level. WKY rats had lower abundance of *Rikenellaceae* but higher abundance of *Alistipes* and *A. putredinis*. *Alistipes*, a commensal belonging to Bacteroidetes, has shown both pathogenic and protective effects in previous studies. Several *Alistipes* species, including *A. putredinis*, can hydrolyze tryptophan to indole (52), thereby competing with the serotonin synthesis pathway from tryptophan. This was confirmed by the negative correlation between colon 5-HT levels and the abundance of *Alistipes* and *A. putredinis* in this study.

The bacteria–fungi interaction differed between WKY rats and Wistar rats, consistent with previous research on depression (26, 27). WKY rats exhibited more correlations between fungi and bacteria compared to Wistar rats. This change contradicted findings in patients with current depressive episodes (27) but aligned with those in patients with multiple sclerosis (53).

The function of the gut microbiota also changes in depression; however, the biomarker pathways of the microbiota remain debated (42, 48, 54). The MetaCyc pathways of WKY rats’ gut bacteria differed from those of Wistar rats. The increases in tyrosine and phenylalanine biosynthesis in WKY rats were consistent with findings in depressive patients (55). Butyrate biosynthesis was increased in WKY rats, contrary to previous findings in MDD patients (42, 48) and depressive rodents (49, 56). The enrichment of butyrate-producing bacteria like *Allobaculum (* 57) and *Butyricimonas (* 58) could be the reason.

The most interesting findings were in the *Lactobacillus* genus. There was less unidentified *Lactobacillus* genus in WKY rats compared to Wistar rats, while the abundance of *L. reuteri* did not differ. NS8 intervention did not affect the abundance of either. However, it significantly increased the abundance of *L. helveticus*. The abundance of the unidentified *Lactobacillus* genus was over 200 times higher than that of *L. helveticus*, and the abundance of *L. reuteri* was six times higher than that of *L. helveticus* in WKY rats. This indicates that not all *Lactobacillus* species can improve depression. This finding could provide insights into previous contradictory results regarding changes in the abundance of *Lactobacillus* in depressive individuals (40, 41, 46) and the inconsistent results of probiotic interventions (25, 44). NS8 supplementation inhibited *Bilophila* in WKY rats. *Bilophila*, an opportunistic pathogen in the Desulfovibrionaceae family that can convert taurine into the toxic metabolite hydrogen sulfide, showed a positive correlation with depressive-like behaviors in this study. It was more abundant in MDD patients (50) and was reduced following *Lactobacillus plantarum* CCFM405 intervention in Parkinson’s disease (PD) mouse models (59).

The only fungus altered by NS8 was Debaryomycetaceae. NS8 supplementation inhibited Debaryomycetaceae in WKY rats, while restraint stress promoted them. The reduction in Debaryomycetaceae due to NS8 intervention was more pronounced in Wistar rats than in WKY rats, but the increase in Debaryomycetaceae due to restraint stress was more obvious in WKY rats than in Wistar rats. However, the study of this fungus is just starting (60). *Candida*, a genus belonging to Debaryomycetaceae, increased in depression, autism spectrum disorders, attention-deficit/hyperactivity, multiple sclerosis, and IBS, and it was associated with increased gut permeability (27, 53, 61–63). Further studies are needed to elucidate the role of *Candida* in WKY rats.

In our previous study, 1 month of live NS8 intervention altered gut microbiota of C57BL/6 J mice (33). In the present study, NS8 supplementation significantly altered bacterial β-diversity and function in Wistar rats but did not reach a significant level in WKY rats. NS8 intervention increased the abundance of *Allobaculum* and *Butyricimonas* in Wistar rats but not in WKY rats. The increase in *L. helveticus* in Wistar rats was much greater than in WKY rats. All these findings suggested that the microbiota of WKY rats was more resistant to NS8 intervention compared to that of Wistar rats, indicating the impact of host genetic context on gut microbiota (43, 64).

### Endogenous depression, monoaminergic system, and *L. helveticus* NS8

The serotonergic system is a major focus of depression research. TPH is the rate-limiting enzyme in serotonin biosynthesis. TPH2 synthesizes 5-HT in the brain and enteric nervous system, while TPH1 synthesizes 5-HT in the gut epithelial cells. Tryptophan is also metabolized via the kynurenine pathway, catalyzed by IDO1 in the brain and gut (65). The present study observed a hypo-serotonergic system, especially in the gut of WKY rats compared to Wistar rats. Deficits in the serotonergic system of WKY rats have been reported before, with the focus mainly on the brain (30, 66). Similarly, hypo-serotonergic systems in both the brain and gut have been reported in other depression models. The TPH2-R439H mouse, an analogous mutation to the R441H mutation found in human depressive patients, not only lacked TPH2 but also exhibited increased depression and anxiety-like behaviors, reduced levels of 5-HT and 5-HIAA in the brain (67), and fewer enteric neuronal 5-HT in the gut (68). The reduction in colon 5-HT and TPH1 was also observed in mice subjected to chronic defeat stress (69) and chronic unpredictable mild stress (70).

The host serotonergic system is closely affected by gut microbiota (65, 71). The higher TPH1 expression in the ileum of WKY rats and lower expression in the colon were both correlated with bacterial butyrate biosynthesis. Reigstad et al. (2016) found that butyrate promoted TPH1 transcription at low concentrations in a human-derived enterochromaffin cell model but inhibited it at high concentrations (72). Considering the unchanged L-tryptophan biosynthesis and higher TPH1 expression in the ileum of WKY rats, the lower 5-HT content in the ileum could possibly be attributed to an increase in bacteria that metabolize tryptophan, such as *A. putredinis* (52). The positive correlation between *L. helveticus* abundance and 5-HIAA content in the hippocampus and colon suggests a potential mechanism by which NS8 enhances the serotonergic system. This regulatory role aligns with its effects observed in exogenous depression model (24) and hyperammonemia model (32). Other probiotics like certain species of *Lactobacillus paracasei* also regulate the serotonergic system in depression models (73).

Catecholamines, including NE and DA, are important monoamine neurotransmitters in both the central and peripheral nervous systems. The noradrenergic systems were elevated in the brain and gut but reduced in the serum of WKY rats. Previous studies have also reported hyperactivity of the brain noradrenergic system in WKY rats (74, 75), but the higher NE content in the colon is reported here for the first time. Meanwhile, NE levels in the colon of mice subjected to chronic unpredictable mild stress were found to be increased (70, 76). Since the mRNA expression of TH decreased in the colon of WKY rats, the increase in NE was probably related to the increased synthesis of L-tyrosine and L-phenylalanine by gut bacteria. Given the significant role of catecholamines in inter-kingdom signaling between the host and microbiota (77), the close association between the microbiota and the host catecholamine system is not unexpected. NS8 intervention reduced NE content in the hypothalamus of WKY rats and NE metabolism in the colon and ileum. The reduction in NE levels by NS8 was likely related to its inhibition of *Bilophila*, a bacterium correlated with NE content in both the colon and serum. The effect of NS8 on the noradrenergic system of WKY rats differed from its effects on Wistar rats observed in this study and SD rats in prior study (24). NS8 treatment increased hippocampal dopamine (DA) content, aligning with previous reports on probiotics’ effects on depression (73, 78). The decreased brain noradrenergic activity and enhanced dopaminergic activity were also observed in the effects of ketamine on WKY rats (79).

### Endogenous depression, neuroendocrine system, and *L. helveticus* NS8

HPA axis dysfunction also plays a key role in depression. CRH, a 41-amino-acid residue peptide, is the dominant regulator of the HPA axis. It is released from the hypothalamus and transported to the anterior pituitary, where it stimulates ACTH secretion. ACTH, in turn, stimulates the synthesis and release of glucocorticoids (cortisol in humans, CORT in rodents) from the adrenal cortex. By binding to GR, endogenous glucocorticoids serve as potent negative regulators of HPA axis activity, including exerting a negative effect on CRH expression (80). The dysfunction of the HPA axis in WKY rats is evident in the present study. We found a higher hippocampal CRH concentration but a decreased circulating ACTH concentration in WKY rats. Increased brain CRH levels are common in MDD patients (80) and WKY rats (30, 81). Additionally, a decrease in serum ACTH content, indicating a blunted ACTH response to CRH, was also found in previous research on MDD (80) and WKY rats (82). Serum CORT levels were also reduced in WKY rats, which contradicts some research (83) but aligns with findings from a chronic corticosterone-induced mouse depression model (73). Considering the positive association between basal glucocorticoids and physical activity in the present study and previous reports (84), the lower locomotor activity levels observed in WKY rats were closely related to their lower CORT levels.

Since the host HPA axis and CRH system are partially regulated by gut microbiota (85, 86), the inconsistency could possibly be attributed to the particular microbiota of WKY rats. The NS8 intervention decreased CRH levels and increased GR levels in the hippocampus, consistent with previous antidepressant treatments (80). The positive effects of NS8 on the HPA axis were consistent with our previous studies (24, 33). Other probiotics, such as *L. paracasei* and *Lactobacillus fermentum*, have also demonstrated their potential to regulate the HPA axis (73, 87).

NPY, a 36-amino-acid peptide, plays a key role in orchestrating endocrine, behavioral, and gastrointestinal responses to stress, similar to CRH (88). NPY expression was also reduced in the colon of WKY rats, consistent with findings in IBS patients with diarrhea (89) and in ulcerative colitis patients and mice (90). Considering the potent orexigenic effect of NPY (91), the slower weight gain of WKY rats was probably associated with reduced NPY expression.

Apart from the above neurotransmitters and neuropeptides, the glutamatergic-GABAergic system also plays a significant role in gut health (92). The GABA content in both the colon and ileum was decreased in WKY rats, a reduction also observed in patients with IBS and diarrhea (93). It was not unreasonable that NS8 intervention increased the GLU content in the ileum, considering a previous study found that 16% of lactic acid bacteria strains isolated from Asian fermented foods are GLU producers (94).

### Endogenous depression, gut barrier impairment, immune abnormalities, and SCFAs

Gut barrier impairment is common in MDD (95) and IBS patients (96). The increased LPS content in the serum and decreased expression of Muc3 and occludin in the colon suggest a compromised gut barrier in WKY rats. Occludin, a transmembrane protein that constitutes TJs, contains the binding site for ZO-1 and is associated with their localization at the TJs (97). The reduction in intestinal occludin was also observed in MDD (98) and IBS (96). Muc3, a transmembrane mucin, was also found to be reduced in deoxycholic acid (DCA)-induced IBS rat models (99, 100). *M. schaedleri*, a putative mucin-degrading species mainly colonizing the mucus layer, was found to increase in colitis, PD, stress-related disorders (101), and in the present endogenous depression. This bacterium could be associated with the above mucus defect, and its abundance decreased with NS8 intervention (significantly in Wistar rats but not significantly in WKY rats). Muc2, the major gel-forming mucin, was decreased in the DCA-induced IBS model (99, 100) but did not change (even slightly increased) in WKY rats, consistent with previously reported goblet cell hyperplasia (the major mucus-secreting cell) in WKY rats (102). Additionally, the increase in LPS and butyrate could be another reason, as both have been shown to increase Muc2 expression in previous research (103).

Chronic inflammation is another characteristic of depression (19). Compared to Wistar rats, WKY rats exhibited higher systemic inflammation but lower immune activation in the gut. The higher circulatory inflammation and LPS content are consistent with previous depression studies (19, 24, 104), whereas the lower immune activation is a new finding. Despite the inconsistencies, the positive correlations between gut proinflammatory cytokines and IDO1, 5-HT, and CRH are consistent with previous research (105–107). The reduced expression of TLR4 (52% decrease) and NF-κB (41% decrease) in the colon could be one reason. TLR4, an innate immune receptor recognizing signals from microorganisms or damaged cells, plays a significant role in neuropsychiatric diseases including depression (108). LPS can activate the TLR4/NF-κB pathway and induce the secretion of a series of proinflammatory cytokines (109). Thus, the higher inflammation in the serum and the lower immune activation in the gut of WKY rats were not surprising. Upregulation of the TLR4/NF-κB pathway is common in individuals with depression (108), whereas TLR4 deletion or knockout alleviates neuroinflammation and behavioral abnormalities in mice (110, 111). Hence, the downregulation of the TLR4/NF-κB pathway possibly protected WKY rats from further immune impairment. Compared to the ileum, which exhibited unchanging expression of TLR4 and NF-κB, the colon showed decreased TLR4 and NF-κB expression and demonstrated much lower immune activation.

The immune status is also closely related with SCFAs (37). By activating targeted receptors such as GPR41 and GPR43, or inhibiting histone deacetylase (HDAC), butyrate and propionate inhibit the secretion of proinflammatory cytokines. Although the SCFA receptors were sharply reduced in the gut of WKY rats, the biosynthesis of butyrate and propionate from bacteria obviously increased. Butyrate also inhibits the immune activation induced by LPS in both the periphery and brain (112). Besides gut immune activation, the normal immune status of the hippocampus (unchanged cytokines and BDNF levels) in WKY rats could also be partially attributed to butyrate.

Although NS8 intervention did not decrease brain inflammation (32) or enhance gut immunity (34, 35) as earlier reported, it did elevate hippocampal BDNF levels in WKY and Wistar rats in the current study. The beneficial effect of NS8 on hippocampal neurogenesis is consistent with our previous findings (24) and other probiotics research on depression (73). NS8 likely improves behaviors by modulating the HPA axis, neurotransmitter metabolism, and neurogenesis in WKY rats, similar to its effects in stressed SD rats (24, 32).

### Genetic context and gut microbiota

Not only do host genetics regulate microbiota composition (43), but the microbiota also contribute to host adaptation and health (5, 64). The deficiency of butyrate-producing bacteria has been regarded as an important characteristic of MDD (42), and butyrate treatment has shown antidepressant effects in exogenous depression (113, 114). However, in hypertension studies, when WKY rats are used as normotensive controls for SHR rats, the results differ significantly. Compared to SHR rats, WKY rats exhibit greater synthesis of butyrate and acetate, lower levels of colon TLR4, lower plasma NE content, better gut barrier integrity, and reduced gut inflammation (115, 116). SHR rats that acquired microbiota from WKY rats through fecal microbiota transplant or cross-fostering not only experienced reduced blood pressure but also alleviated most of the abnormalities. Conversely, WKY rats that received microbiota from SHR rats exhibited the opposite effects. Furthermore, the magnitude of these changes was influenced by both gut microbiota and genetic context (115, 117–119). Compared with earlier reports, the reduced hippocampal BDNF content (120) and the hypercortisolism (83) were not observed in the present study. Based on the above results, we speculate that the gut microbiota of WKY rats may have developed compensatory mechanisms for host genomic deficits over hundreds of generations, including the enrichment of butyrate-producing bacteria and enhanced butyrate synthesis. Previous studies have demonstrated the hypotensive, anti-inflammatory, and neuroprotective effects of WKY rats’ gut microbiota; further studies are needed to elucidate the antidepressant effects. Nonetheless, the effects of the genetic context on gut microbiota cannot be ignored. The resistance of WKY rats to the effects of NS8 intervention and cross-fostering compared with SD rats also indicated this (24, 121).

### Limitations

Under similar NS8 intervention conditions, the improvements in behavior and the microbiota–gut–brain axis in WKY rats were less pronounced than those previously reported in exogenous models without genetic defects (24, 32–35). The combination of *L. rhamnosus* HN001 and Lipid 70 induced a more pronounced shift in the gut microbiota of WKY rats compared to HN001 alone (45). Additionally, combining *L. fermentum* NS9 with anthocyanidin better alleviates sodium iodate-induced retinal degeneration than anthocyanidin alone (122). Two months of NS8 intervention had more significant impact on the brain peptidome of mice than 1 month (33), and the complex probiotics *L. helveticus* NS8 and *L. fermentum* NS9 demonstrated significant antidepressant and antianxiety effects in our unpublished clinical study. Extending the intervention duration or combining with other probiotics and/or prebiotics may result in further improvements. Moreover, more in-depth animal studies and human research are needed to elucidate the molecular mechanisms and clinical effects of *L. helveticus* NS8.

## Conclusion

In conclusion, WKY rats exhibited typical depressive behaviors and harbored specific gut bacteria and mycobiota. The depression phenotype of WKY rats was not only attributed to their genetic context but also closely related to their gut microbiota. Microbiota–gut–brain dysfunction was evident in WKY rats. The hypo-serotonergic system, hyper-noradrenergic system, defective HPA axis, compromised gut barrier integrity, increased systemic inflammation, dampened gut immunity, and abnormal gut microbiota collectively contributed to the specific depression phenotype. One month of *L. helveticus* NS8 intervention partially restored behaviors, the serotonergic system, the dopaminergic and noradrenergic system, the HPA axis, and the microbiota. The findings of this study could be promising for patients, especially those with a familial predisposition to depression, treatment-resistant depression, and/or combined with IBS symptoms.

## Data Availability

The datasets presented in this study can be found in online repositories. The names of the repository/repositories and accession number(s) can be found below: PRJNA1074102 and PRJNA1074118 (SRA).

## Supplementary material

Supplementary Figure 1 WKY rats presented abnormalities with Wistar rats in behaviors in experiment one. **(A)** showed the brief flow diagram of experiment one. **(B)** showed the body weight gain of the rats during the experiment one. **(C)** showed the immobility time and climbing time in forced swimming test, and **(D)** showed the locomotor activities in open field test. ScheirerRayHare test followed by Dunn’s Test adjusted by **(B–H)** method was used in **(C)**, Two-way ANOVA followed by Tukey HSD test was used in **(D)**, and repeated measures ANOVA was used in B. RS, restraint stress.

Supplementary Figure 2 The β-diversity of gut microbiota in experiment 1 and the effects of NS8 intervention on gut-brain axis-related bacterial MetaCyc pathways in experiment 2. **(A, E)** showed the principal coordinates analysis (PCOA) of bacteria and **(B)** showed the principal component analysis (PCA) of the predicted function of bacteria. **(C)** presented the PCOA of fungi and **(D)** presented the PCA of the predicted function of fungi. **(A-E)** showed the results in experiment 1 and F-O showed the results in experiment 2. F showed the synthesis of lipopolysaccharide from bacteria and G showed the synthesis of peptidoglycan. H-K showed the synthesis of four key amino acid from bacteria, including L- tryptophan, L-tyrosine, L-phenylalanine, and L-glutamate and L-glutamine. L-O showed the synthesis of short chain fatty acids from bacteria, including butyrate, propionate, acetate, and lactate. ScheirerRayHare test followed by Dunn’s Test adjusted by **(B–H)** method was used.

Supplementary Figure 3 The correlation network between physiological biomarkers. The network showed the correlation between physiological biomarkers, and the locomotor activity was also included in this analysis. The blue lines were negative correlation while the red lines were positive correlation. The thicker the line, the greater the absolute value of the correlation coefficient. Spearman’s correlations were used and only the adjusted p-value (**B–H** method) <0.05 (Benjamini–Hochberg method) and absolute r >0.4 were showed.

Supplementary Table 1 Sensitivity of the ELISA kits.

Supplementary Table 2 Primer sequences for RT-PCR.

Supplementary Table 3 The behaviors and alpha diversity in experiment one.

Supplementary Table 4 The behaviors and alpha diversity in experiment two.

Supplementary Table 5 The brain-gut axis indicators in experiment two.

## Glossary

MDD: major depressive disorder
HPA axis: hypothalamic–pituitary–adrenal axis
BDNF: brain-derived neurotrophic factor
5-HT: 5-hydroxytryptamine/serotonin
WKY rat: Wistar–Kyoto rat
SD rat: Sprague–Dawley rat
SHR rat: spontaneous hypertension rat
OFT: open field locomotor activity test
FST: forced swimming test
SPT: sucrose preference test
IBS: irritable bowel syndrome
E1: experiment one
E2: experiment two
ELISA: enzyme-linked immunosorbent assay
5-HIAA: 5-hydroxyindoleacetic acid
NE: norepinephrine
DA: dopamine
GR: glucocorticoid receptor
CRH: corticotropin-releasing hormone
TPH: tryptophan hydroxylase
TH: tyrosine hydroxylase
IDO: indoleamine 2, 3-dioxygenase
IL-17a: interleukin-17a
IL-10: interleukin-10
ACTH: adrenocorticotropic hormone
CORT: corticosterone
HVA: 4-hydroxy-3-methoxyphenylacetic
GABA: gamma-aminobutyric acid
GLU: glutamic acid
SP: substance P
IL-6: interleukin-6
IL-1β: interleukin-1β
TNF-α: tumor necrosis factor-alpha
TGF-β: transforming growth factor-beta
IFN-γ: interferon-gamma
LPS: lipopolysaccharide
RT-PCR: reverse transcriptase-polymerase chain reaction
TLR4: Toll-like receptor 4
NF-κB: nuclear factor kappa B
NPY: neuropeptide Y
GPR41: free fatty acid receptor 3
GPR43: free fatty acid receptor 2
Olr59: olfactory receptor 59
ZO-1: zonula occludens-1/tight junction protein 1
Muc2: Mucin-2
Muc3: Mucin-3
GAPDH: glyceraldehyde-3-phosphate dehydrogenase
PCOA: principal coordinates analysis
PCA: principal component analysis
TJs: tight junctions
SCFAs: short-chain fatty acids
PD: Parkinson’s disease
HDAC: histone deacetylase

## References

[1] Collaborators* GMD. Global, regional, and national burden of 12 mental disorders in 204 countries and territories, 1990–2019: a systematic analysis for the Global Burden of Disease Study 2019. Lancet Psychiatry. (2022) 9:137–50. doi: 10.1016/S2215-0366(21)00395-3 PMC8776563 35026139

[2] Liang S WU X Wang T Hu X Jin F. Recognizing depression from the microbiota–gut–brain axis. Int J Mol Sci. (2018) 19:E1592. doi: 10.3390/ijms19061592 PMC6032096 29843470

[3] Valles-Colomer M Falony G Darzi Y Tigchelaar EF Wang J Tito RY. The neuroactive potential of the human gut microbiota in quality of life and depression. Nat Microbiol. (2019) 4:623–32. doi: 10.1038/s41564-018-0337-x 30718848

[4] Chang L Wei Y Hashimoto K. Brain-gut-microbiota axis in depression: A historical overview and future directions. Brain Res Bull. (2022) 182:44–56. doi: 10.1016/j.brainresbull.2022.02.004 35151796

[5] Liang S Wu X Jin F. Gut-brain psychology: rethinking psychology from the microbiota-gut-brain axis. Front Integr Neurosci. (2018) 12:33. doi: 10.3389/fnint.2018.00033 30271330 PMC6142822

[6] Zheng P Zeng B Zhou C Liu M Fang Z Xu X. Gut microbiome remodeling induces depressive-like behaviors through a pathway mediated by the host’s metabolism. Mol Psychiatry. (2016) 21:786–96. doi: 10.1038/mp.2016.44 27067014

[7] Kelly JR Borre Y O’Brien C Patterson E El Aidy S Deane J. Transferring the blues: Depression-associated gut microbiota induces neurobehavioural changes in the rat. J Psychiatr Res. (2016) 82:109–18. doi: 10.1016/j.jpsychires.2016.07.019 27491067

[8] Green JE Berk M Mohebbi M Loughman A McGuinness AJ Castle D. Feasibility, acceptability, and safety of faecal microbiota transplantation in the treatment of major depressive disorder: A pilot randomized controlled trial. Can J Psychiatry. (2023) 68:315–26. doi: 10.1177/07067437221150508 PMC10192831 36637229

[9] Doll JPK Vazquez-Castellanos JF Schaub AC Schweinfurth N Kettelhack C Schneider E. Fecal microbiota transplantation (FMT) as an adjunctive therapy for depression-case report. Front Psychiatry. (2022) 13:815422. doi: 10.3389/fpsyt.2022.815422 35250668 PMC8891755

[10] Hayer SS Hwang S Clayton JB. Antibiotic-induced gut dysbiosis and cognitive, emotional, and behavioral changes in rodents: a systematic review and meta-analysis. Front Neurosci. (2023) 17:1237177. doi: 10.3389/fnins.2023.1237177 37719161 PMC10504664

[11] Pouranayatihosseinabad M Bezabih Y Hawrelak J Peterson GM Veal F Mirkazemi C. Antibiotic use and the development of depression: A systematic review. J Psychosom Res. (2023) 164:111113. doi: 10.1016/j.jpsychores.2022.111113 36502554

[12] Liu RT Walsh RFL Sheehan AE. Prebiotics and probiotics for depression and anxiety: A systematic review and meta-analysis of controlled clinical trials. Neurosci Biobehav Rev. (2019) 102:13–23. doi: 10.1016/j.neubiorev.2019.03.023 31004628 PMC6584030

[13] Wallace CJK Milev R. The effects of probiotics on depressive symptoms in humans: a systematic review. Ann Gen Psychiatry. (2017) 16:14. doi: 10.1186/s12991-017-0138-2 28239408 PMC5319175

[14] Paiva IHR Duarte-Silva E Peixoto CA. The role of prebiotics in cognition, anxiety, and depression. Eur Neuropsychopharmacol. (2020) 34:1–18. doi: 10.1016/j.euroneuro.2020.03.006 32241688

[15] Aslam H Green J Jacka FN Collier F Berk M Pasco J. Fermented foods, the gut and mental health: a mechanistic overview with implications for depression and anxiety. Nutr Neurosci. (2020) 23:659–71. doi: 10.1080/1028415X.2018.1544332 30415609

[16] Lassale C Batty GD Baghdadli A Jacka F Sánchez-Villegas A Kivimäki M. Healthy dietary indices and risk of depressive outcomes: a systematic review and meta-analysis of observational studies. Mol Psychiatry. (2018) 24:965–86. doi: 10.1038/s41380-018-0237-8 PMC6755986 30254236

[17] Sandhu KV Sherwin E Schellekens H Stanton C Dinan TG Cryan JF. Feeding the microbiota-gut-brain axis: diet, microbiome, and neuropsychiatry. Trans research: J Lab Clin Med. (2017) 179:223–44. doi: 10.1016/j.trsl.2016.10.002 27832936

[18] Tan X Zhang L Wang D Guan S Lu P Xu X. Influence of early life stress on depression: from the perspective of neuroendocrine to the participation of gut microbiota. Aging (Albany NY). (2021) 13:25588–601. doi: 10.18632/aging.v13i23 PMC8714134 34890365

[19] Cruz-Pereira JS Rea K Nolan YM O’Leary OF Dinan TG Cryan JF. Depression’s unholy trinity: dysregulated stress, immunity, and the microbiome. Annu Rev Psychol. (2020) 71:49–78. doi: 10.1146/annurev-psych-122216-011613 31567042

[20] Schroeder BO Birchenough GMH Stahlman M Arike L Johansson MEV Hansson GC. Bifidobacteria or fiber protects against diet-induced microbiota-mediated colonic mucus deterioration. Cell Host Microbe. (2018) 23:27–40 e7. doi: 10.1016/j.chom.2017.11.004 29276171 PMC5764785

[21] Pagliai G Dinu M Madarena MP Bonaccio M Iacoviello L Sofi F. Consumption of ultra-processed foods and health status: a systematic review and meta-analysis. Br J Nutr. (2021) 125:308–18. doi: 10.1017/S0007114520002688 PMC7844609 32792031

[22] Yong SJ Tong T Chew J Lim WL. Antidepressive mechanisms of probiotics and their therapeutic potential. Front Neurosci. (2019) 13:1361. doi: 10.3389/fnins.2019.01361 32009871 PMC6971226

[23] Gao J Zhao L Cheng Y Lei W Wang Y Liu X. Probiotics for the treatment of depression and its comorbidities: A systemic review. Front Cell Infect Microbiol. (2023) 13:1167116. doi: 10.3389/fcimb.2023.1167116 37139495 PMC10149938

[24] Liang S Wang T Hu X Luo J Li W Wu X. Administration of Lactobacillus helveticus NS8 improves behavioral, cognitive, and biochemical aberrations caused by chronic restraint stress. Neuroscience. (2015) 310:561–77. doi: 10.1016/j.neuroscience.2015.09.033 26408987

[25] Yang Z Li J Gui X Shi X Bao Z Han H. Updated review of research on the gut microbiota and their relation to depression in animals and human beings. Mol Psychiatry. (2020) 25:2759–72. doi: 10.1038/s41380-020-0729-1 32332994

[26] Hao SR Zhang Z Zhou YY Zhang X Sun WJ Yang Z. Altered gut bacterial-fungal interkingdom networks in children and adolescents with depression. J Affect Disord. (2023) 332:64–71. doi: 10.1016/j.jad.2023.03.086 37003434

[27] Jiang HY Pan LY Zhang X Zhang Z Zhou YY Ruan B. Altered gut bacterial-fungal interkingdom networks in patients with current depressive episode. Brain behavior. (2020) 10:e01677. doi: 10.1002/brb3.1677 32533650 PMC7428472

[28] Tejani-Butt S Kluczynski J Pare WP. Strain-dependent modification of behavior following antidepressant treatment. Prog Neuro-Psychoph. (2003) 27:7–14. doi: 10.1016/S0278-5846(02)00308-1 12551720

[29] Nam H Clinton SM Jackson NL Kerman IA. Learned helplessness and social avoidance in the Wistar-Kyoto rat. Front Behav Neurosci. (2014) 8:109. doi: 10.3389/fnbeh.2014.00109 24744709 PMC3978372

[30] Millard SJ Weston-Green K Newell KA. The Wistar-Kyoto rat model of endogenous depression: A tool for exploring treatment resistance with an urgent need to focus on sex differences. Prog Neuropsychopharmacol Biol Psychiatry. (2020) 101:109908. doi: 10.1016/j.pnpbp.2020.109908 32145362

[31] Hyland NP O’Mahony SM O’Malley D O’Mahony CM Dinan TG Cryan JF. Early-life stress selectively affects gastrointestinal but not behavioral responses in a genetic model of brain-gut axis dysfunction. Neurogastroenterol Motil. (2015) 27:105–13. doi: 10.1111/nmo.12486 25443141

[32] Luo J Wang T Liang S Hu X Li W Jin F. Ingestion of Lactobacillus strain reduces anxiety and improves cognitive function in the hyperammonemia rat. Sci China Life Sci. (2014) 57:327–35. doi: 10.1007/s11427-014-4615-4 24554471

[33] Zhang P Wu X Liang S Shao X Wang Q Chen R. A dynamic mouse peptidome landscape reveals probiotic modulation of the gut-brain axis. Sci Signal. (2020) 13:1–14. doi: 10.1126/scisignal.abb0443 32723811

[34] Rong J Zheng H Liu M Hu X Wang T Zhang X. Probiotic and anti-inflammatory attributes of an isolate Lactobacillus helveticus NS8 from Mongolian fermented koumiss. BMC Microbiol. (2015) 15:196. doi: 10.1186/s12866-015-0525-2 26428623 PMC4591576

[35] Rong J Liu S Hu C Liu C. Single probiotic supplement suppresses colitis-associated colorectal tumorigenesis by modulating inflammatory development and microbial homeostasis. J Gastroenterol Hepatol. (2019) 34:1182–92. doi: 10.1111/jgh.14516 30357910

[36] Chiba S Numakawa T Ninomiya M Richards MC Wakabayashi C Kunugi H. Chronic restraint stress causes anxiety- and depression-like behaviors, downregulates glucocorticoid receptor expression, and attenuates glutamate release induced by brain-derived neurotrophic factor in the prefrontal cortex. Prog Neuro-Psychoph. (2012) 39:112–9. doi: 10.1016/j.pnpbp.2012.05.018 22664354

[37] Dalile B Van Oudenhove L Vervliet B Verbeke K. The role of short-chain fatty acids in microbiota-gut-brain communication. Nat Rev Gastroenterol hepatol. (2019) 16:461–78. doi: 10.1038/s41575-019-0157-3 31123355

[38] Martin-Gallausiaux C Marinelli L Blottiere HM Larraufie P Lapaque N. SCFA: mechanisms and functional importance in the gut. Proc Nutr Soc. (2021) 80:37–49. doi: 10.1017/S0029665120006916 32238208

[39] Overstreet DH. Modeling depression in animal models. Methods Mol Biol. (2012) 829:125–44. doi: 10.1007/978-1-61779-458-2_7 22231810

[40] Nikolova VL Smith MRB Hall LJ Cleare AJ Stone JM Young AH. Perturbations in gut microbiota composition in psychiatric disorders: A review and meta-analysis. JAMA Psychiatry. (2021) 78:1343–54. doi: 10.1001/jamapsychiatry.2021.2573 PMC8444066 34524405

[41] Barandouzi ZA Starkweather AR Henderson WA Gyamfi A Cong XS. Altered composition of gut microbiota in depression: A systematic review. Front Psychiatry. (2020) 11:541. doi: 10.3389/fpsyt.2020.00541 32587537 PMC7299157

[42] McGuinness AJ Davis JA Dawson SL Loughman A Collier F O’Hely M. A systematic review of gut microbiota composition in observational studies of major depressive disorder, bipolar disorder and schizophrenia. Mol Psychiatry. (2022) 27:1920–35. doi: 10.1038/s41380-022-01456-3 PMC9126816 35194166

[43] Bubier JA Chesler EJ Weinstock GM. Host genetic control of gut microbiome composition. Mamm Genome. (2021) 32:263–81. doi: 10.1007/s00335-021-09884-2 PMC8295090 34159422

[44] Alli SR Gorbovskaya I Liu JCW Kolla NJ Brown L Muller DJ. The gut microbiome in depression and potential benefit of prebiotics, probiotics and synbiotics: A systematic review of clinical trials and observational studies. Int J Mol Sci. (2022) 23(9):4494. doi: 10.3390/ijms23094494 PMC9101152 35562885

[45] Dalziel JE Zobel G Dewhurst H Hurst C Olson T Rodriguez-Sanchez R. A diet enriched with lacticaseibacillus rhamnosus HN001 and milk fat globule membrane alters the gut microbiota and decreases amygdala GABA a receptor expression in stress-sensitive rats. Int J Mol Sci. (2023) 24(13):10433. doi: 10.3390/ijms241310433 PMC10341670 37445611

[46] Herselman MF Bailey S Bobrovskaya L. The effects of stress and diet on the “Brain-gut” and “Gut-brain” Pathways in animal models of stress and depression. Int J Mol Sci. (2022) 23(4):2013. doi: 10.3390/ijms23042013 PMC8875876 35216133

[47] Simpson CA Diaz-Arteche C Eliby D Schwartz OS Simmons JG Cowan CSM. The gut microbiota in anxiety and depression - A systematic review. Clin Psychol review. (2020) 83:101943. doi: 10.1016/j.cpr.2020.101943 33271426

[48] Liu RT Rowan-Nash AD Sheehan AE Walsh RFL Sanzari CM Korry BJ. Reductions in anti-inflammatory gut bacteria are associated with depression in a sample of young adults. Brain behavior immunity. (2020) 88:308–24. doi: 10.1016/j.bbi.2020.03.026 PMC7415740 32229219

[49] Tian P O’Riordan KJ Lee YK Wang G Zhao J Zhang H. Towards a psychobiotic therapy for depression: Bifidobacterium breve CCFM1025 reverses chronic stress-induced depressive symptoms and gut microbial abnormalities in mice. Neurobiol stress. (2020) 12:100216. doi: 10.1016/j.ynstr.2020.100216 32258258 PMC7109524

[50] Caso JR MacDowell KS Gonzalez-Pinto A Garcia S de Diego-Adelino J Carceller-Sindreu M. Gut microbiota, innate immune pathways, and inflammatory control mechanisms in patients with major depressive disorder. Trans Psychiatry. (2021) 11:645. doi: 10.1038/s41398-021-01755-3 PMC8692500 34934041

[51] Dalziel JE Fraser K Young W McKenzie CM Bassett SA Roy NC. Gastroparesis and lipid metabolism-associated dysbiosis in Wistar-Kyoto rats. Am J Physiol Gastrointestinal liver Physiol. (2017) 313:G62–72. doi: 10.1152/ajpgi.00008.2017 PMC5538835 28408641

[52] Parker BJ Wearsch PA Veloo ACM Rodriguez-Palacios A. The genus alistipes: gut bacteria with emerging implications to inflammation, cancer, and mental health. Front Immunol. (2020) 11:906. doi: 10.3389/fimmu.2020.00906 32582143 PMC7296073

[53] Yadav M Ali S Shrode RL Shahi SK Jensen SN Hoang J. Multiple sclerosis patients have an altered gut mycobiome and increased fungal to bacterial richness. PloS One. (2022) 17:e0264556. doi: 10.1371/journal.pone.0264556 35472144 PMC9041819

[54] Lai WT Deng WF Xu SX Zhao J Xu D Liu YH. Shotgun metagenomics reveals both taxonomic and tryptophan pathway differences of gut microbiota in major depressive disorder patients. Psychol Med. (2021) 51:90–101. doi: 10.1017/S0033291719003027 31685046

[55] Park S Li C Wu X Zhang T. Gut microbiota alterations and their functional differences in depression according to enterotypes in Asian individuals. Int J Mol Sci. (2023) 24(17):13329. doi: 10.3390/ijms241713329 PMC10487633 37686135

[56] Wu M Tian T Mao Q Zou T Zhou CJ Xie J. Associations between disordered gut microbiota and changes of neurotransmitters and short-chain fatty acids in depressed mice. Trans Psychiatry. (2020) 10:350. doi: 10.1038/s41398-020-01038-3 PMC7567879 33067412

[57] Balakrishnan B Luckey D Bodhke R Chen J Marietta E Jeraldo P. Prevotella histicola protects from arthritis by expansion of allobaculum and augmenting butyrate production in humanized mice. Front Immunol. (2021) 12:609644. doi: 10.3389/fimmu.2021.609644 34017324 PMC8130672

[58] Sakamoto M Takagaki A Matsumoto K Kato Y Goto K Benno Y. Butyricimonas synergistica gen. nov., sp. nov. and Butyricimonas virosa sp. nov., butyric acid-producing bacteria in the family ‘Porphyromonadaceae’ isolated from rat faeces. Int J Syst Evol Microbiol. (2009) 59:1748–53. doi: 10.1099/ijs.0.007674-0 19542124

[59] Chu C Yu L Li Y Guo H Zhai Q Chen W. Lactobacillus plantarum CCFM405 against Rotenone-Induced Parkinson’s Disease Mice via Regulating Gut Microbiota and Branched-Chain Amino Acids Biosynthesis. Nutrients. (2023) 15(7):1737. doi: 10.3390/nu15071737 PMC10096885 37049578

[60] Vitenberg T Opatovsky I. Assessing fungal diversity and abundance in the black soldier fly and its environment. J Insect Sci. (2022) 22(6):3. doi: 10.1093/jisesa/ieac066 PMC9673256 36398851

[61] Strati F Cavalieri D Albanese D De Felice C Donati C Hayek J. New evidences on the altered gut microbiota in autism spectrum disorders. Microbiome. (2017) 5:24. doi: 10.1186/s40168-017-0242-1 28222761 PMC5320696

[62] Wang LJ Li SC Yeh YM Lee SY Kuo HC Yang CY. Gut mycobiome dysbiosis and its impact on intestinal permeability in attention-deficit/hyperactivity disorder. J Child Psychol Psychiatry. (2023) 64:1280–91. doi: 10.1111/jcpp.13779 37016804

[63] van Thiel I de Jonge W van den Wijngaard R. Fungal feelings in the irritable bowel syndrome: the intestinal mycobiome and abdominal pain. Gut Microbes. (2023) 15:2168992. doi: 10.1080/19490976.2023.2168992 36723172 PMC9897793

[64] Suzuki TA Ley RE. The role of the microbiota in human genetic adaptation. Science. (2020) 370(6521):eaaz6827. doi: 10.1126/science.aaz6827 33273073

[65] Agus A Planchais J Sokol H. Gut microbiota regulation of tryptophan metabolism in health and disease. Cell Host Microbe. (2018) 23:716–24. doi: 10.1016/j.chom.2018.05.003 29902437

[66] Yamada M Kawahara Y Kaneko F Kishikawa Y Sotogaku N Poppinga WJ. Upregulation of the dorsal raphe nucleus-prefrontal cortex serotonin system by chronic treatment with escitalopram in hyposerotonergic Wistar-Kyoto rats. Neuropharmacology. (2013) 72:169–78. doi: 10.1016/j.neuropharm.2013.04.044 23643754

[67] Beaulieu J-M Zhang X Rodriguiz RM Sotnikova TD Cools MJ Wetsel WC. Role of GSK3β in behavioral abnormalities induced by serotonin deficiency. Proc Natl Acad Sci United States America. (2008) 105:1333–8. doi: 10.1073/pnas.0711496105 PMC2234138 18212115

[68] Israelyan N Del Colle A Li Z Park Y Xing A Jacobsen JPR. Effects of serotonin and slow-release 5-hydroxytryptophan on gastrointestinal motility in a mouse model of depression. Gastroenterology. (2019) 157:507–21 e4. doi: 10.1053/j.gastro.2019.04.022 31071306 PMC6650329

[69] Xie R Jiang P Lin L Jiang J Yu B Rao J. Oral treatment with Lactobacillus reuteri attenuates depressive-like behaviors and serotonin metabolism alterations induced by chronic social defeat stress. J Psychiatr Res. (2020) 122:70–8. doi: 10.1016/j.jpsychires.2019.12.013 31927268

[70] Wang L Sun Y Zhao T Li Y Zhao X Zhang L. Antidepressant effects and mechanisms of the total iridoids of valeriana jatamansi on the brain-gut axis. Planta Med. (2020) 86:172–9. doi: 10.1055/a-1068-9686 31801162

[71] Yano JM Yu K Donaldson GP Shastri GG Ann P Ma L. Indigenous bacteria from the gut microbiota regulate host serotonin biosynthesis. Cell. (2015) 161:264–76. doi: 10.1016/j.cell.2015.02.047 PMC4393509 25860609

[72] Reigstad CS Salmonson CE Rainey JF 3rd Szurszewski JH Linden DR Sonnenburg JL. Gut microbes promote colonic serotonin production through an effect of short-chain fatty acids on enterochromaffin cells. FASEB J. (2015) 29:1395–403. doi: 10.1096/fj.14-259598 PMC4396604 25550456

[73] Wei CL Wang S Yen JT Cheng YF Liao CL Hsu CC. Antidepressant-like activities of live and heat-killed Lactobacillus paracasei PS23 in chronic corticosterone-treated mice and possible mechanisms. Brain Res. (2019) 1711:202–13. doi: 10.1016/j.brainres.2019.01.025 30684456

[74] Bruzos-Cidon C Miguelez C Rodriguez JJ Gutierrez-Lanza R Ugedo L Torrecilla M. Altered neuronal activity and differential sensitivity to acute antidepressants of locus coeruleus and dorsal raphe nucleus in Wistar Kyoto rats: a comparative study with Sprague Dawley and Wistar rats. Eur Neuropsychopharmacol. (2014) 24:1112–22. doi: 10.1016/j.euroneuro.2014.02.007 24582527

[75] El Mansari M Hamoudeh R Daniels S Blier P. Wistar Kyoto rats exhibit decreased serotonin neuronal firing and increased norepinephrine burst activity but dampened hippocampal alpha(2)-adrenoceptor sensitivity. J Psychopharmacol. (2023) 37:1105–15. doi: 10.1177/02698811231209235 37942525

[76] Feng S Meng C Liu Y Yi Y Liang A Zhang Y. Bacillus licheniformis prevents and reduces anxiety-like and depression-like behaviours. Appl Microbiol Biotechnol. (2023) 107:4355–68. doi: 10.1007/s00253-023-12580-7 37209162

[77] Sarkodie EK Zhou S Baidoo SA Chu W. Influences of stress hormones on microbial infections. Microb Pathog. (2019) 131:270–6. doi: 10.1016/j.micpath.2019.04.013 30981718

[78] Rehman MU Ghazanfar S Ul Haq R Ullah S Khan S Wu J. Probiotics (Bacillus clausii and Lactobacillus fermentum NMCC-14) Ameliorate Stress Behavior in Mice by Increasing Monoamine Levels and mRNA Expression of Dopamine Receptors (D(1) and D(2)) and Synaptophysin. Front Pharmacol. (2022) 13:915595. doi: 10.3389/fphar.2022.915595 35928261 PMC9343877

[79] Daniels S El Mansari M Hamoudeh R Blier P. Ketamine promptly normalizes excess norepinephrine and enhances dopamine neuronal activity in Wistar Kyoto rats. Front Pharmacol. (2023) 14:1276309. doi: 10.3389/fphar.2023.1276309 38026921 PMC10644068

[80] Naughton M Dinan TG Scott LV. Corticotropin-releasing hormone and the hypothalamic–pituitary–adrenal axis in psychiatric disease. Handb Clin Neurol. (2014) 124:69–91. doi: 10.1016/B978-0-444-59602-4.00005-8 25248580

[81] Shepard JD Myers DA. Strain differences in anxiety-like behavior: association with corticotropin-releasing factor. Behav Brain Res. (2008) 186:239–45. doi: 10.1016/j.bbr.2007.08.013 17904655

[82] Hauger RL Shelat SG Redei EE. Decreased corticotropin-releasing factor receptor expression and adrenocorticotropic hormone responsiveness in anterior pituitary cells of Wistar-Kyoto rats. J neuroendocrinol. (2002) 14:126–34. doi: 10.1046/j.0007-1331.2001.00752.x 11849372

[83] Malkesman O Braw Y Malayan R Weizman A Overstreet DH Shabat-Simon M. Two different putative genetic animal models of childhood depression. Biol Psychiatry. (2006) 59:17–23. doi: 10.1016/j.biopsych.2005.05.039 16095569

[84] Singleton JM Garland T Jr. Influence of corticosterone on growth, home-cage activity, wheel running, and aerobic capacity in house mice selectively bred for high voluntary wheel-running behavior. Physiol Behav. (2019) 198:27–41. doi: 10.1016/j.physbeh.2018.10.001 30292826

[85] Huo R Zeng B Zeng L Cheng K Li B Luo Y. Microbiota modulate anxiety-like behavior and endocrine abnormalities in hypothalamic-pituitary-adrenal axis. Front Cell Infect Microbiol. (2017) 7:489. doi: 10.3389/fcimb.2017.00489 29250490 PMC5715198

[86] Liu S Guo R Liu F Yuan Q Yu Y Ren F. Gut microbiota regulates depression-like behavior in rats through the neuroendocrine-immune-mitochondrial pathway. Neuropsychiatr Dis Treat. (2020) 16:859–69. doi: 10.2147/NDT.S243551 PMC7127849 32280227

[87] Wang T Hu X Liang S Li W Wu X Wang L. Lactobacillus fermentum NS9 restores the antibiotic induced physiological and psychological abnormalities in rats. Beneficial Microbes. (2015) 6:707–17. doi: 10.3920/BM2014.0177 25869281

[88] Rana T Behl T Sehgal A Singh S Sharma N Abdeen A. Exploring the role of neuropeptides in depression and anxiety. Prog Neuropsychopharmacol Biol Psychiatry. (2022) 114:110478. doi: 10.1016/j.pnpbp.2021.110478 34801611

[89] Rasmusson AM. The gut peptide neuropeptide Y and post-traumatic stress disorder. Curr Opin Endocrinol Diabetes Obes. (2017) 24:3–8. doi: 10.1097/MED.0000000000000301 27898588

[90] Joo MK Lee JW Woo JH Kim HJ Kim DH Choi JH. Regulation of colonic neuropeptide Y expression by the gut microbiome in patients with ulcerative colitis and its association with anxiety- and depression-like behavior in mice. Gut Microbes. (2024) 16:2319844. doi: 10.1080/19490976.2024.2319844 38404132 PMC10900276

[91] Patel HR Qi Y Hawkins EJ Hileman SM Elmquist JK Imai Y. Neuropeptide Y deficiency attenuates responses to fasting and high-fat diet in obesity-prone mice. Diabetes. (2006) 55:3091–8. doi: 10.2337/db05-0624 17065347

[92] Mazzoli R Pessione E. The neuro-endocrinological role of microbial glutamate and GABA signaling. Front Microbiol. (2016) 7:1934. doi: 10.3389/fmicb.2016.01934 27965654 PMC5127831

[93] Aggarwal S Ahuja V Paul J. Dysregulation of GABAergic signalling contributes in the pathogenesis of diarrhea-predominant irritable bowel syndrome. J Neurogastroenterol motility. (2018) 24:422–30. doi: 10.5056/jnm17100 PMC6034664 29852727

[94] Zareian M Ebrahimpour A Bakar FA Mohamed AKS Forghani B Ab-Kadir MSB. A glutamic acid-producing lactic acid bacteria isolated from Malaysian fermented foods. Int J Mol Sci. (2012) 13:5482–97. doi: 10.3390/ijms13055482 PMC3382744 22754309

[95] Stevens BR Goel R Seungbum K Richards EM Holbert RC Pepine CJ. Increased human intestinal barrier permeability plasma biomarkers zonulin and FABP2 correlated with plasma LPS and altered gut microbiome in anxiety or depression. Gut. (2018) 67:1555–7. doi: 10.1136/gutjnl-2017-314759 PMC5851874 28814485

[96] Hanning N Edwinson AL Ceuleers H Peters SA De Man JG Hassett LC. Intestinal barrier dysfunction in irritable bowel syndrome: a systematic review. Therap Adv Gastroenterol. (2021) 14:1756284821993586. doi: 10.1177/1756284821993586 PMC7925957 33717210

[97] Barbara G Barbaro MR Fuschi D Palombo M Falangone F Cremon C. Inflammatory and microbiota-related regulation of the intestinal epithelial barrier. Front Nutr. (2021) 8:718356. doi: 10.3389/fnut.2021.718356 34589512 PMC8475765

[98] Liu P Liu Z Wang J Wang J Gao M Zhang Y. Immunoregulatory role of the gut microbiota in inflammatory depression. Nat Commun. (2024) 15:3003. doi: 10.1038/s41467-024-47273-w 38589368 PMC11001948

[99] Rodriguez-Nogales A Algieri F Vezza T Garrido-Mesa J Molina-Tijeras JA Rodriguez-Cabezas ME. Calcium pyruvate exerts beneficial effects in an experimental model of irritable bowel disease induced by DCA in rats. Nutrients. (2019) 11(1):140. doi: 10.3390/nu11010140 PMC6356508 30634696

[100] Ruiz-Malagon AJ Rodriguez-Sanchez MJ Rodriguez-Sojo MJ Vezza T Pischel I Algieri F. Intestinal anti-inflammatory and visceral analgesic effects of a Serpylli herba extract in an experimental model of irritable bowel syndrome in rats. Front Pharmacol. (2022) 13:967644. doi: 10.3389/fphar.2022.967644 36120292 PMC9479127

[101] Herp S Durai Raj AC Salvado Silva M Woelfel S Stecher B. The human symbiont Mucispirillum schaedleri: causality in health and disease. Med Microbiol Immunol. (2021) 210:173–9. doi: 10.1007/s00430-021-00702-9 PMC7615636 34021796

[102] O’Malley D Julio-Pieper M Gibney SM Dinan TG Cryan JF. Distinct alterations in colonic morphology and physiology in two rat models of enhanced stress-induced anxiety and depression-like behaviour. Stress. (2010) 13:114–22. doi: 10.3109/10253890903067418 20214436

[103] Paone P Cani PD. Mucus barrier, mucins and gut microbiota: the expected slimy partners? Gut. (2020) 69:2232–43. doi: 10.1136/gutjnl-2020-322260 PMC7677487 32917747

[104] Desbonnet L Garrett L Clarke G Kiely B Cryan JF Dinan TG. Effects of the probiotic Bifidobacterium infantis in the maternal separation model of depression. Neuroscience. (2010) 170:1179–88. doi: 10.1016/j.neuroscience.2010.08.005 20696216

[105] Lashgari NA Roudsari NM Shayan M Niazi Shahraki F Hosseini Y Momtaz S. IDO/Kynurenine; novel insight for treatment of inflammatory diseases. Cytokine. (2023) 166:156206. doi: 10.1016/j.cyto.2023.156206 37120946

[106] Najjar SA Hung LY Margolis KG. Serotonergic control of gastrointestinal development, motility, and inflammation. Compr Physiol. (2023) 13:4851–68. doi: 10.1002/cphy.c220024 PMC10373054 37358510

[107] Baritaki S de Bree E Chatzaki E Pothoulakis C. Chronic stress, inflammation, and colon cancer: A CRH system-driven molecular crosstalk. J Clin Med. (2019) 8(10):1669. doi: 10.3390/jcm8101669 PMC6833069 31614860

[108] Garcia Bueno B Caso JR Madrigal JL Leza JC. Innate immune receptor Toll-like receptor 4 signalling in neuropsychiatric diseases. Neurosci Biobehav Rev. (2016) 64:134–47. doi: 10.1016/j.neubiorev.2016.02.013 26905767

[109] Ghosh SS Wang J Yannie PJ Ghosh S. Intestinal barrier dysfunction, LPS translocation, and disease development. J Endocr Soc. (2020) 4:bvz039. doi: 10.1210/jendso/bvz039 32099951 PMC7033038

[110] Fei X Dou YN Lv W Ding B Wei J Wu X. TLR4 deletion improves cognitive brain function and structure in aged mice. Neuroscience. (2022) 492:1–17. doi: 10.1016/j.neuroscience.2022.04.007 35405301

[111] Perez-Pardo P Dodiya HB Engen PA Forsyth CB Huschens AM Shaikh M. Role of TLR4 in the gut-brain axis in Parkinson’s disease: a translational study from men to mice. Gut. (2019) 68:829–43. doi: 10.1136/gutjnl-2018-316844 30554160

[112] Yao Y Cai X Fei W Ye Y Zhao M Zheng C. The role of short-chain fatty acids in immunity, inflammation and metabolism. Crit Rev Food Sci Nutr. (2022) 62(1):1–12. doi: 10.1080/10408398.2020.1854675 33261516

[113] Yamawaki Y Yoshioka N Nozaki K Ito H Oda K Harada K. Sodium butyrate abolishes lipopolysaccharide-induced depression-like behaviors and hippocampal microglial activation in mice. Brain Res. (2018) 1680:13–38. doi: 10.1016/j.brainres.2017.12.004 29229502

[114] Sun J Wang F Hong G Pang M Xu H Li H. Antidepressant-like effects of sodium butyrate and its possible mechanisms of action in mice exposed to chronic unpredictable mild stress. Neurosci Lett. (2016) 618:159–66. doi: 10.1016/j.neulet.2016.03.003 26957230

[115] Toral M Robles-Vera I de la Visitacion N Romero M Yang T Sanchez M. Critical role of the interaction gut microbiota - sympathetic nervous system in the regulation of blood pressure. Front Physiol. (2019) 10:231. doi: 10.3389/fphys.2019.00231 30930793 PMC6423906

[116] Robles-Vera I Toral M de la Visitacion N Sanchez M Gomez-Guzman M Romero M. Probiotics prevent dysbiosis and the rise in blood pressure in genetic hypertension: role of short-chain fatty acids. Mol Nutr Food Res. (2020) 64:e1900616. doi: 10.1002/mnfr.201900616 31953983

[117] Adnan S Nelson JW Ajami NJ Venna VR Petrosino JF Bryan RM Jr.. Alterations in the gut microbiota can elicit hypertension in rats. Physiol Genomics. (2017) 49:96–104. doi: 10.1152/physiolgenomics.00081.2016 28011881 PMC5336599

[118] Abboud FM Cicha MZ Ericsson A Chapleau MW Singh MV. Altering early life gut microbiota has long-term effect on immune system and hypertension in spontaneously hypertensive rats. Front Physiol. (2021) 12:752924. doi: 10.3389/fphys.2021.752924 34777016 PMC8586697

[119] Nelson JW Phillips SC Ganesh BP Petrosino JF Durgan DJ Bryan RM. The gut microbiome contributes to blood-brain barrier disruption in spontaneously hypertensive stroke prone rats. FASEB J. (2021) 35:e21201. doi: 10.1096/fj.202001117R 33496989 PMC8238036

[120] Vinod KY Xie S Psychoyos D Hungund BL Cooper TB Tejani-Butt SM. Dysfunction in fatty acid amide hydrolase is associated with depressive-like behavior in Wistar Kyoto rats. PloS One. (2012) 7:e36743. doi: 10.1371/journal.pone.0036743 22606285 PMC3351478

[121] Howells FM Bindewald L Russell VA. Cross-fostering does not alter the neurochemistry or behavior of spontaneously hypertensive rats. Behav Brain functions: BBF. (2009) 5:24. doi: 10.1186/1744-9081-5-24 19549323 PMC2711096

[\122] Xing Y Liang S Zhang L Ni H Zhang X Wang J. Combination of Lactobacillus fermentum NS9 and aronia anthocyanidin extract alleviates sodium iodate-induced retina degeneration. Sci Rep. (2023) 13:8380. doi: 10.1038/s41598-023-34219-3 37225720 PMC10209211

