# Supplementary material for: Supplementation with Lactobacillus helveticus NS8 alleviated behavioral, neural, endocrine, and microbiota abnormalities in an endogenous rat model of depression
Source: Front Immunol. 2024 Sep 13;15:1407620. doi: 10.3389/fimmu.2024.1407620 (PMC11428200; doi:10.3389/fimmu.2024.1407620)

**Supplemental figures**

**Figure 1. WKY rats presented abnormalities with Wistar rats in behaviors in experiment one.** A showed the brief flow diagram of experiment one. B showed the body weight gain of the rats during the experiment one. C showed the immobility time and climbing time in forced swimming test, and D showed the locomotor activities in open field test. ScheirerRayHare test followed by Dunn’s Test adjusted by B–H method was used in C, Two-way ANOVA followed by Tukey HSD test was used in D, and repeated measures ANOVA was used in B. RS, restraint stress.


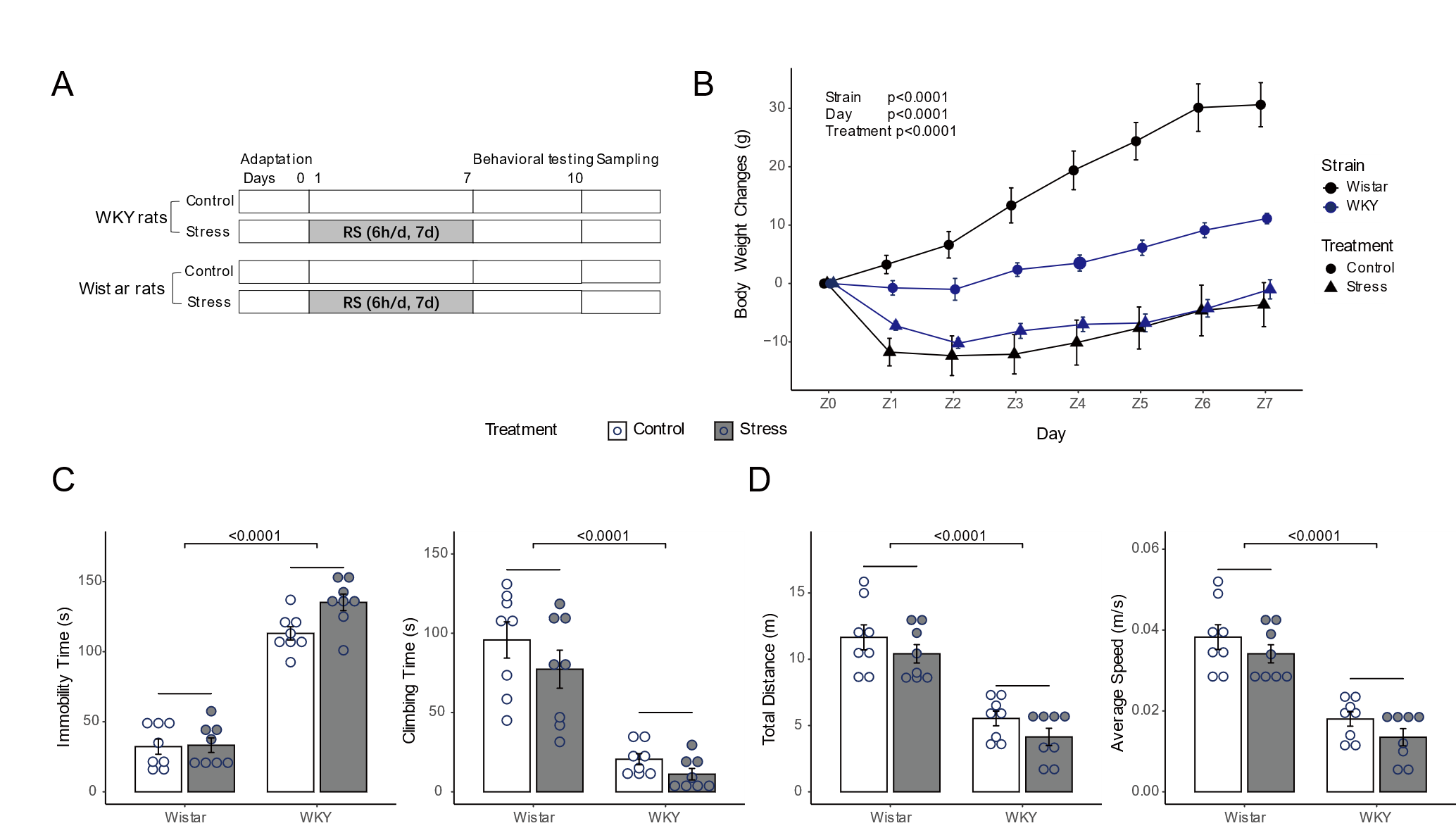


**Figure 2. The β-diversity of gut microbiota in experiment 1 and the effects of NS8 intervention on gut-brain axis-related bacterial MetaCyc pathways in experiment 2.** A and E showed the principal coordinates analysis (PCOA) of bacteria and B showed the principal component analysis (PCA) of the predicted function of bacteria. C presented the PCOA of fungi and D presented the PCA of the predicted function of fungi. A-E showed the results in experiment 1 and F-O showed the results in experiment 2. F showed the synthesis of lipopolysaccharide from bacteria and G showed the synthesis of peptidoglycan. H-K showed the synthesis of four key amino acid from bacteria, including L-tryptophan, L-tyrosine, L-phenylalanine, and L-glutamate and L-glutamine. L-O showed the synthesis of short chain fatty acids from bacteria, including butyrate, propionate, acetate, and lactate. ScheirerRayHare test followed by Dunn’s Test adjusted by B–H method was used.


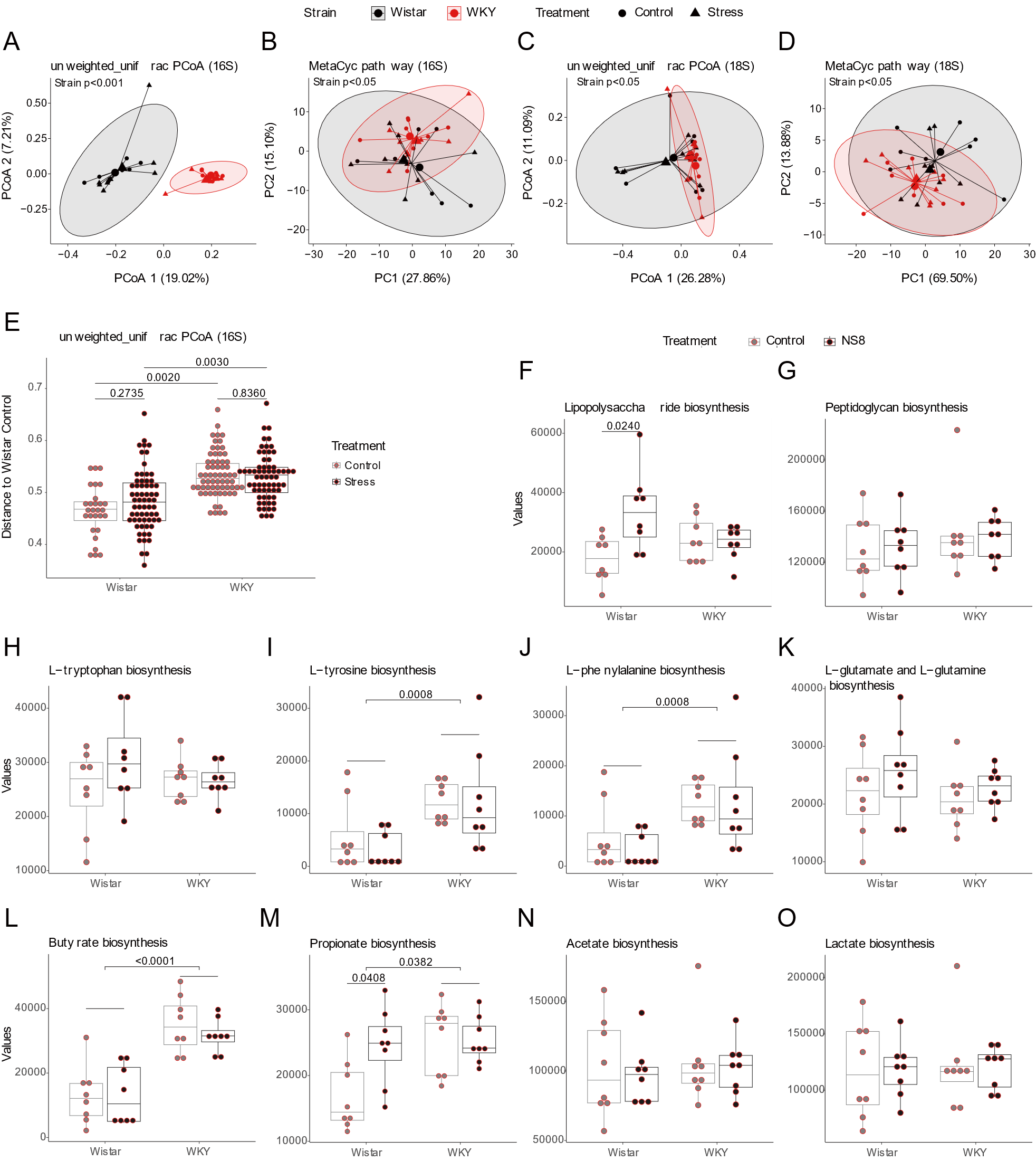


**Figure 3. The correlation network between physiological biomarkers.** The network showed the correlation between physiological biomarkers, and the locomotor activity was also included in this analysis. The blue lines were negative correlation while the red lines were positive correlation. The thicker the line, the greater the absolute value of the correlation coefficient. Spearman’s correlations were used and only the adjusted p-value (B–H method) <0.05 (Benjamini–Hochberg method) and absolute r >0.4 were showed.


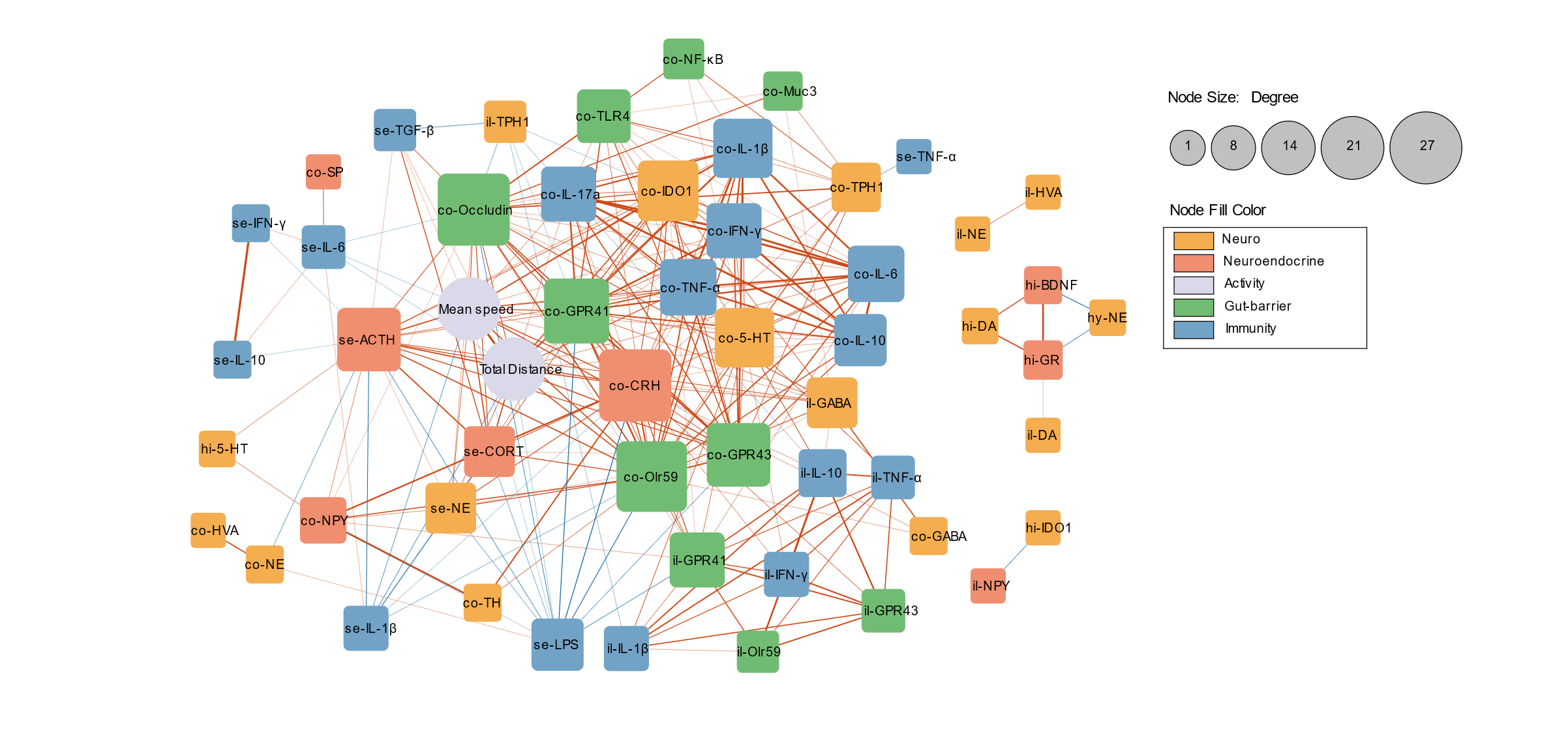

Supplement: Supplementary Figure 1 — WKY rats presented abnormalities with Wistar rats in behaviors in experiment one. (A) showed the brief flow diagram of experiment one. (B) showed the body weight gain of the rats during the experiment one. (C) showed the immobility time and climbing time in forced swimming test, and (D) showed the locomotor activities in open field test. ScheirerRayHare test followed by Dunn’s Test adjusted by (B–H) method was used in (C), Two-way ANOVA followed by Tukey HSD test was used in (D), and repeated measures ANOVA was used in B. RS, restraint stress. [file DataSheet1.docx]
